# JUNB‐FBXO21‐ERK axis promotes cartilage degeneration in osteoarthritis by inhibiting autophagy

**DOI:** 10.1111/acel.13306

**Published:** 2021-01-15

**Authors:** Zhiming Lin, Jianing Miao, Tao Zhang, Ming He, Ziyuan Wang, Xinyuan Feng, Lunhao Bai

**Affiliations:** ^1^ Department of Orthopedics Shengjing Hospital of China Medical University Shenyang China; ^2^ Medical Research Center/Liaoning Key Laboratory of Research and Application of Animal Models for Environmental and Metabolic Diseases Shenyang China; ^3^ Department of Thoracic Surgery Xiamen Branch of Zhongshan Hospital of Fudan University Xiamen China

**Keywords:** autophagy, cartilage degeneration, ERK, FBXO21, JUNB, metabolism, osteoarthritis, rats

## Abstract

Osteoarthritis (OA) is a heterogeneous disease that is extremely hard to cure owing to its complex regulation network of pathogenesis, especially cartilage degeneration. FBXO21 is a subunit of ubiquitin E3 ligases that degrades P‐glycoprotein and EID1 by ubiquitination and activates the JNK and p38 pathways; however, its role in OA remains unknown. Here, the main objective of this study was to evaluate the potential effects and mechanism of FBXO21 in OA degeneration, we revealed that FBXO21 is upregulated in the cartilage of patients with OA, aging, and monosodium iodoacetate‐induced OA rats, and chondrocytes treated with interleukin‐1β, tumor necrosis factor‐α, and lipopolysaccharide. Moreover, the in vivo and in vitro knockdown of FBXO21 suppressed OA‐related cartilage degeneration, as evidenced by activated autophagy, upregulated anabolism, alleviated apoptosis, and downregulated catabolism. In contrast, its overexpression promoted OA‐related cartilage degeneration. In addition, using mass spectrometry and co‐immunoprecipitation assay, we demonstrated that the downstream mechanism of FBXO21 inhibits autophagy by interacting with and phosphorylating ERK. Furthermore, FBXO21 alleviated anabolism and enhanced apoptosis and catabolism by inhibiting autophagy in rat chondrocytes. Interestingly, for its upstream mechanism, JUNB promoted FBXO21 expression by directly targeting the FBXO21 promoter, thus further accelerating cartilage degeneration in SW1353 cells and rat chondrocytes. Overall, our findings reveal that the JUNB‐FBXO21‐ERK axis regulates OA apoptosis and cartilage matrix metabolism by inhibiting autophagy. Therefore, FBXO21 is an attractive target for regulating OA pathogenesis, and its knockdown may provide a novel targeted therapy for OA.

## Graphical abstract

FBXO21 is upregulated in osteoarthritis (OA). Knockdown and overexpression of FBXO21 suppresses and promotes OA‐related degeneration, respectively. Mechanistically, FBXO21 inhibits autophagy by interacting with and phosphorylating ERK, and enhances OA‐related degeneration by inhibiting autophagy. JUNB regulates FBXO21 expression via targeting FBXO21 promoter and accelerates OA‐related degeneration by promoting FBXO21.

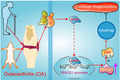

## 1 INTRODUCTION

Osteoarthritis (OA) is a considerably heterogeneous disease that is associated with age, gender, obesity, and joint injury (Kraus et al., 2015; Loeser et al., 2016) and affects approximately 240 million people worldwide, resulting in a great economic burden to the society (Hunter & Bierma‐Zeinstra, 2019; Liu et al., 2018). OA is mainly characterized by subchondral bone remodeling, joint inflammation, osteophyte formation, and cartilage degeneration (Guan et al., 2018; Ji et al., 2019; Zhang et al., 2017) that is influenced by cell stress and cartilage matrix metabolism (Glyn‐Jones et al., 2015). OA pathogenesis involves elevated apoptosis and increased catabolism and decreased anabolism in cartilage matrix metabolism (Kim et al., 2014; Rahmati et al., 2017). Autophagy depends on the progression of OA (Lotz & Caramés, 2011). However, to date, there remains no disease‐modifying treatment of OA. Therefore, it is of utmost importance to elucidate its pathogenesis, which can yield effective therapeutic strategies.

It has been reported that the F‐box protein family members, including FBXO32 and FBXO6 (Kim et al., 2017; Wang et al., 2020), are related to OA. FBXO21, a member of the F‐box protein family, is a subunit of the Skp1‐cullin‐F‐box (SCF) ubiquitin E3 ligases, which acts on phosphorylation‐dependent ubiquitination degradation. Normally, FBXO21 degrades the P‐glycoprotein and EID1 by ubiquitination (Ravindranath et al., 2015; Yoshida et al., 2015; Zhang et al., 2015). Dramatically, FBXO21 activates apoptosis signal‐regulating kinase 1 (ASK1), instead of degrading, resulting in the activation of JNK and p38 pathways during antiviral innate response (Yu et al., 2016). However, its biological role in OA remains unknown and has not been studied.

JUNB is a transcription factor (TF) containing a basic leucine zipper and belongs to the JUN family that includes JUND and c‐JUN. JUNB is elevated in human chondrocytes and cartilage with OA and mice cartilage with OA. In addition, JUNB can bind to the promoter of the matrix metalloproteinase13 (MMP13) that promotes the expression of MMP13 and reduces that of type II collagen (COL2A1) after the activation by cytokines such as interleukin (IL)‐1β (Rhee et al., 2017). We have previously found binding sites of JUNB in the FBXO21 (*Homo sapiens*) promoter using the JASPAR database (Fornes et al., 2020); thus, we hypothesized that JUNB functions by targeting FBXO21.

ERK is a serine/threonine protein kinase that can be activated by cytokines in OA (Latourte et al., 2017). ERK is related to cartilage calcification and osteophyte formation but has little effect on cartilage matrix metabolism (Prasadam et al., 2013). It is mainly involved in regulating cell proliferation, apoptosis (Prasadam et al., 2010; Zhou et al., 2019), and autophagy (Corcelle et al., 2007). Autophagy is a mechanism of cell self‐protection that acts as an intracellular scavenger to maintain intracellular homeostasis and widely occurs in eukaryotic cells (Kang & Elledge, 2016). Autophagy is a double‐edged sword that may be beneficial and detrimental. In OA, autophagy increases in the early stage but decreases in the late stage (Li et al., 2016). After we showed that ERK interacts with FBXO21 in a preliminary experiment, we hypothesized that FBXO21 may function by targeting ERK and autophagy.

In this study, the main objective was to evaluate the potential effects and mechanism of FBXO21 in OA degeneration. We firstly demonstrated FBXO21 accumulated in articular cartilage of patients with knee OA, articular cartilage of Sprague Dawley (SD) rats with OA of aging and monosodium iodoacetate (MIA) model, and chondrocytes with OA. Then, we investigated underlying mechanism that could regulate cartilage degeneration in OA. Subsequently, we identified the involvement of the JUNB‐FBXO21‐ERK axis in regulating apoptosis, anabolism, and catabolism of OA by inhibiting autophagy. To the best of our knowledge, this study is the first to explore the biological role of FBXO21 in OA, which sought to fill the gap in literature. Taken together, these results indicate that the novel functions of FBXO21 might provide a new therapeutic avenue for OA.

## 2 RESULTS

### 2.1 FBXO21 is upregulated in the damaged area of articular cartilage in patients with knee OA and associated with clinicopathological features

**FIGURE 1:**
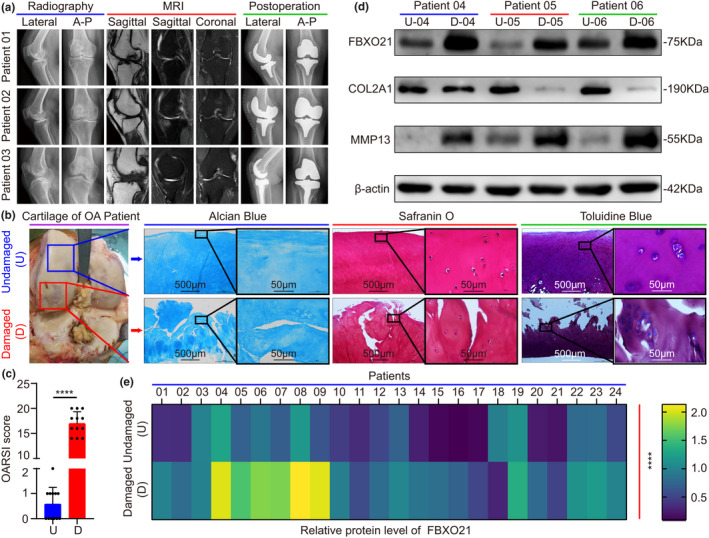
Expression of FBXO21 is upregulated in the damaged area of articular cartilage in patients with knee osteoarthritis (OA) who underwent total knee replacement. (a) Plain radiograph and magnetic resonance imaging (MRI) scans (*n* = 3). A–P: anterior–posterior. (b) Alcian Blue, Safranin O, and Toluidine Blue staining and (c) the Osteoarthritis Research Society International (OARSI) score of undamaged (U) and damaged (D) cartilage tissues of patients with OA (*n* = 12). Blue boxes and arrows represent undamaged (U) cartilage, where those in red represent damaged (D) cartilage. Black box represents those at higher magnification. (d) Immunoblotting of FBXO21, COL2A1, and MMP13 and (e) their quantification with heat maps for relative protein level of FBXO21 in undamaged (U) and damaged (D) areas, with β‐actin as the endogenous control (*n* = 24). U‐04: undamaged (U) area of patient‐04; D‐04: damaged (D) area of patient‐04. The ages of the patients 04‐06 are 68, 75, and 73 years old. Data are presented as the mean ± SD; ****p < 0.0001

To evaluate the potential role of FBXO21 in OA, we first demonstrated the validity of the articular cartilage samples divided into undamaged (U) and damaged (D) area from 24 patients with knee OA by collecting clinical images (Figure 1a), performing Alcian Blue, Safranin O, Toluidine Blue staining and assigning Osteoarthritis Research Society International (OARSI) score. We observed that undamaged cartilage appeared to be smooth, whereas damaged ones were characterized by cracks and fissures (Figure 1b,c). Next, the relative protein levels of FBXO21, COL2A1, and MMP13 were detected by immunoblotting. FBXO21 was significantly upregulated in damaged cartilage of patients with knee OA (Figure 1d,e).

To identify clinical significance of FBXO21 level in OA, relationships among patient characteristics, including age, gender, obesity gradation, Kellgren–Lawrence gradation, and FBXO21 expression in the damaged area are presented using a Sankey diagram (Figure S1a,b). Among 24 patients with OA, 10 had low FBXO21 levels, whereas 14 had high FBXO21 levels in the damaged cartilage (Table 1). Chi‐square test results revealed that high FBXO21 expression was associated with Kellgren–Lawrence gradation (*p* = 0.045) (Table 2). Spearman correlation analysis confirmed that high FBXO21 expression correlated with Body Mass Index (BMI; *p* = 0.004), obesity gradation (*p* = 0.012), and Kellgren–Lawrence gradation (*p* = 0.001) (Table S1). Hence, these results indicate that FBXO21 is upregulated in the damaged area of articular cartilage in patients with knee OA and is associated with clinicopathological features, including Kellgren–Lawrence gradation, BMI, and obesity gradation.

**TABLE 1:** Baseline characteristics of patients with osteoarthritis (*n* = 24)

Characteristics	Number of cases (%)
Age (years)	
<65	7 (29.2)
≥65	17 (70.8)
Gender	
Male	4 (16.7)
Female	20 (83.3)
Obesity gradation^a^	
Underweight	3 (12.5)
Normal weight	5 (20.8)
Overweight	7 (29.2)
Obesity	9 (37.5)
Kellgren–Lawrence gradation	
III	11 (45.8)
IV	13 (54.2)
Expression of FBXO21^b^	
Low expression	10 (41.7)
High expression	14 (58.3)

^a^ Underweight: BMI < 18.5; Normal weight: 18.5 ≤ BMI < 24; Overweight: 24 ≤ BMI < 28; Obesity: BMI ≥ 28.

^b^ The maximal difference (eleventh minus tenth is 0.0889 in ascending order) near median was used to classify between the low expression or high expression of FBXO21 instead of the median (thirteenth minus twelfth is 0.0076 in ascending order).

**TABLE 2:** Correlation between FBXO21 expression and the baseline characteristics of patients with osteoarthritis (*n* = 24)

Characteristics	FBXO21 expression		*P* *
	Low	High	
Age (years)			
<65	4	3	0.324
≥65	6	11	
Gender			
Male	3	1	0.139
Female	7	13	
Obesity gradation			
Underweight	2	1	0.413
Normal weight	2	3	
Overweight	4	3	
Obesity	2	7	
Kellgren–Lawrence gradation			
III	7	4	0.045
IV	3	10	

* *P* values were analyzed using chi‐square test with *p* < 0.05 as significant.

### 2.2 FBXO21 accumulates in rat articular cartilage and chondrocytes with OA

**FIGURE 2:**
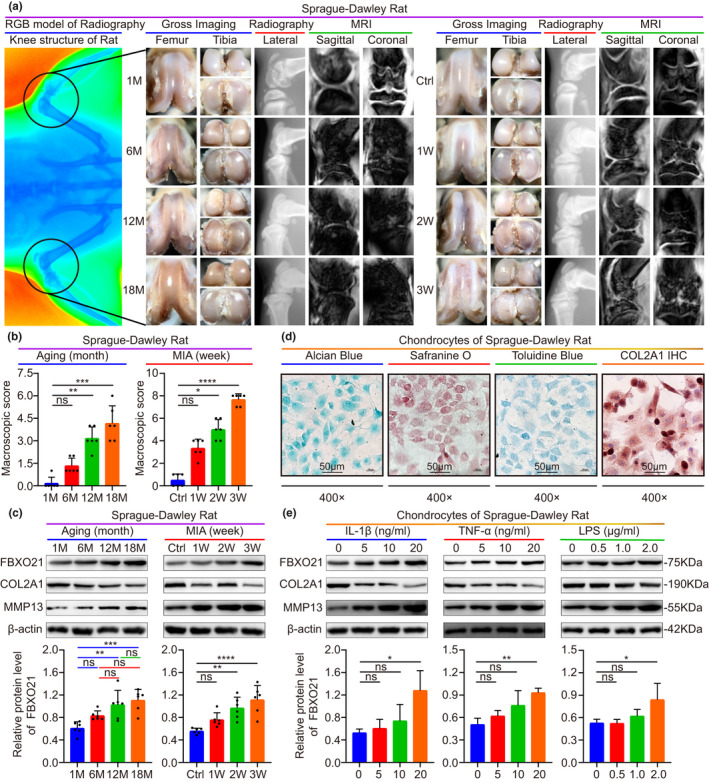
FBXO21 accumulates in rat articular cartilage and chondrocytes with OA. (a) Gross imaging, plain radiograph, and magnetic resonance imaging (MRI) scans from aging (middle) and monosodium iodoacetate (MIA) (right) OA rat models. Radiography presents the whole knee structure (left). M: Month; Ctrl: Saline Controls; W: Week. (b) Macroscopic score. Immunoblotting (upper) results of FBXO21, COL2A1, and MMP13 and the quantification (lower) of FBXO21, with β‐actin as the endogenous control, in the (c) aging and MIA OA rat models and in (e) chondrocytes stimulated with interleukin (IL)‐1β, tumor necrosis factor (TNF)‐α, and lipopolysaccharide (LPS). (d) Identification of rat chondrocytes using Alcian Blue, Safranin O, or Toluidine Blue and immunohistochemistry (IHC) staining of COL2A1. Data are presented as the mean ± SD; ns: not significant, *p < 0.05, **p < 0.01, ***p < 0.001, ****p < 0.0001

In the aging and MIA‐induced rat OA models, OA model was confirmed by gross imaging, plain radiograph, magnetic resonance imaging (MRI), and macroscopic score of gross imaging. We observed increased surface roughening, fibrillation, fissures, and erosions down to subchondral bone during OA aggravation. There were significant differences in the 12 month (M), 18 M, MIA 2 week (W), and MIA 3 W groups compared with control (Figure 2a,b). The relative protein levels of FBXO21 gradually elevated in the aging and MIA‐induced OA models and showed statistical significance in the 12 M, 18 M, 2 W, and 3 W groups (Figure 2c). To further identify expression of FBXO21 in primary chondrocytes of rats and SW1353 cells with OA, primary chondrocytes of rats were confirmed by Alcian Blue, Safranin O, Toluidine Blue, and COL2A1 immunohistochemistry (IHC) staining. Positively stained cells for COL2A1 were observed (Figure 2d). Similar observations were noted in terms of the relative protein levels of FBXO21, COL2A1, and MMP13 in rat primary chondrocytes (Figure 2e) and SW1353 cells (Figure S2b) in all three OA models induced by IL‐1β, tumor necrosis factor (TNF)‐α, and lipopolysaccharide (LPS) treatments. Statistical significance was observed compared with control in the following treatment groups: 20 ng/ml of IL‐1β, 20 ng/ml of TNF‐α, 2 μg/ml of LPS in rat chondrocytes and 10 and 20 ng/ml of TNF‐α and 1 μg/ml of LPS in SW1353 cells.

Furthermore, we found that FBXO21 level was enhanced in rat chondrocytes of IL‐1β OA model based on the results of immunofluorescence analysis (Figure S2a). We also observed that during aging, the expression of COL2A1 was decreased, whereas that of FBXO21 was increased, showing statistical significance in 6 M, 12 M, and 18 M groups for COL2A1, and 12 M and 18 M groups for FBXO21 compared with 1 M control (Figure S2c–f). Taken together, these results show that FBXO21 accumulates in both articular cartilage and chondrocytes with OA.

### 2.3 FBXO21 knockdown suppresses OA‐related degeneration in MIA‐treated rats and IL‐1β‐treated rat chondrocytes

**FIGURE 3:**
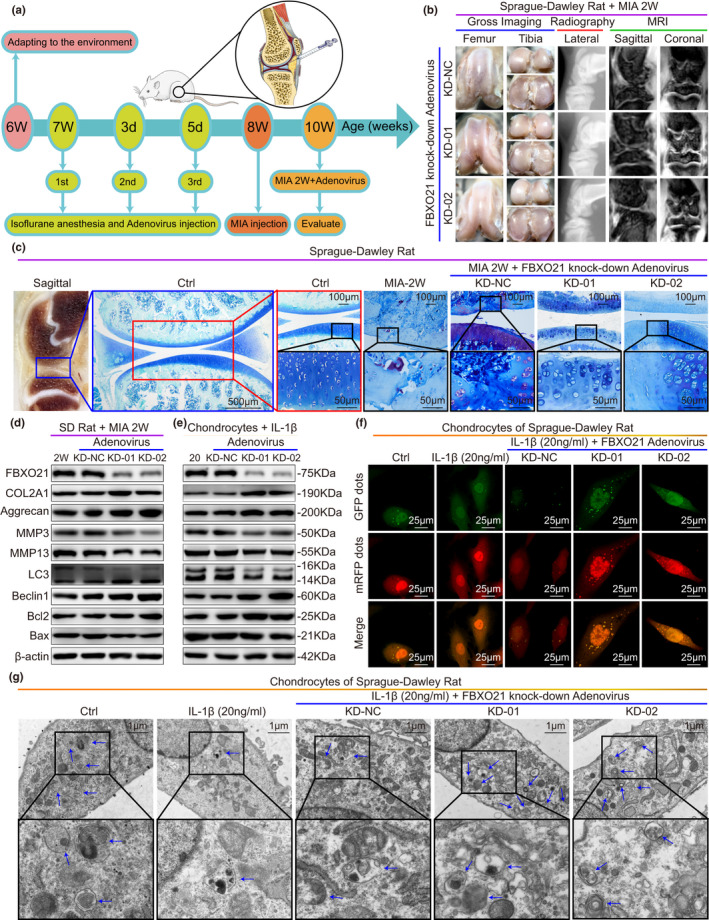
FBXO21 knockdown suppresses OA‐related degeneration in MIA‐treated rats and IL‐1β‐treated rat chondrocytes. (a) Experimental diagram of the monosodium iodoacetate (MIA) OA rat models treated with adenovirus (Ad) carrying FBXO21‐specific knockdown (KD) shRNA and overexpression (OE) plasmid. Rats were evaluated at age of 10 weeks. 1st: first; 2nd: second; 3rd: third; d: day. (b) Gross imaging, plain radiograph, and MRI scans of MIA‐2W OA rats. KD‐Ad‐shRNA‐NC (KD‐NC), KD‐Ad‐shRNA‐FBXO21‐01 (KD‐01), and KD‐Ad‐shRNA‐FBXO21‐02 (KD‐02) groups were formed. NC: negative control. (c) Toluidine Blue staining of rat knee joints and their sagittal section (left). Boxed regions represent higher magnification. Ctrl: Saline Controls. Immunoblotting results of FBXO21, COL2A1, Aggrecan, MMP3, MMP13, LC3, Beclin1, Bcl2, and Bax, with β‐actin as endogenous control, in (d) rat knee cartilage and (e) rat chondrocytes. 2 W: MIA‐2 W; 20: 20 ng/ml of interleukin (IL)‐1β. (f) mRFP‐GFP‐LC3 adenovirus double label in chondrocytes after knockdown of FBXO21. mRFP (red) indicates autolysosomes (ALs); Merge (yellow) indicates autophagosomes (APs). (g) Ultrastructural features of APs in the chondrocytes observed using transmission electron microscopy. Black box represents higher magnification. Blue arrows indicate APs

We next explored the function of FBXO21 in OA pathogenesis. SD rats were preinjected with either knockdown‐sh‐FBXO21‐01/02 (KD‐01/02) or knockdown‐sh‐Negative control (KD‐NC) adenovirus three times a week into their knee joints one week before the MIA injection. Rats were then sacrificed two weeks after MIA injection as described in the methods section and schematic (Figure 3a). Using gross imaging, plain radiograph, MRI, and macroscopic score of gross imaging, we found decreased surface roughening, fibrillation, fissures, and erosions down to subchondral bone after FBXO21 knockdown, with statistical significance in KD‐01 and KD‐02 groups (Figure 3b, Figure S3a). Toluidine Blue staining, Safranin O staining, and Mankin and OARSI scores of SD rat knee joints revealed significantly alleviated cartilage degradation, higher cartilage thickness, and lower Mankin and OARSI grades after FBXO21 knockdown (Figure 3c, Figures S3b and S4a). To further investigate the role of FBXO21 in OA treatment, chondrocytes of SD rats with OA (stimulated with 20 ng/ml of IL‐1β) were transfected with FBXO21 knockdown adenovirus. Knockdown efficiency of FBXO21 and corresponding phenotype markers in vivo and in vitro were confirmed by quantitative real‐time PCR (qRT‐PCR) and immunoblotting. FBXO21 expression was reduced, whereas the levels of protective markers, including COL2A1, Aggrecan, LC3 II/I, and Beclin 1, were significantly upregulated, except for that of the Bcl2/Bax protein in vitro. Among the destructive markers, the expression of MMP13 was downregulated in vivo, but that of the MMP3 protein was not (Figure 3d,e, Figure S3c–f). mRFP‐GFP‐LC3 adenovirus double label and transmission electron microscopy (TEM) manifested that autolysosomes (ALs) and autophagosomes (APs), in which APs were characterized with double membranes (Klionsky et al., 2012), were upregulated after FBXO21 knockdown (Figure 3f,g, Figure S3g,h). Flow cytometry analysis results showed that apoptosis decreased after FBXO21 knockdown (Figure S3i,j). Together, these results suggest that FBXO21 knockdown suppresses OA‐related degeneration in MIA‐treated rats and IL‐1β‐treated rat chondrocytes.

### 2.4 FBXO21 overexpression promotes OA‐related degeneration in MIA‐treated rats and IL‐1β‐treated rat chondrocytes

**FIGURE 4:**
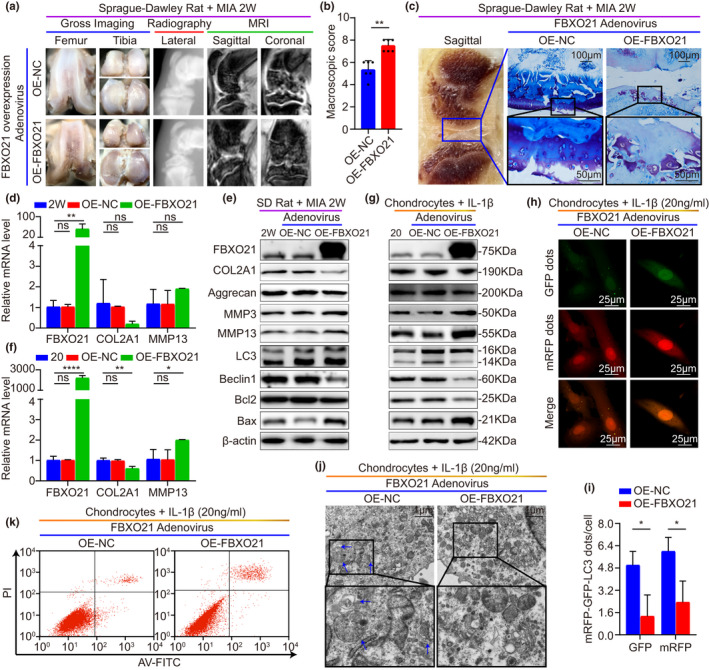
FBXO21 overexpression promotes OA‐related degeneration in MIA‐treated rats and IL‐1β‐treated rat chondrocytes. (a) Gross imaging, plain radiograph, and magnetic resonance imaging (MRI) scans of MIA‐2 W OA rats after transfection with FBXO21 overexpression (OE) adenovirus (Ad) divided into OE‐Ad‐NC (OE‐NC) and OE‐Ad‐FBXO21‐3xflag (OE‐FBXO21). NC: negative control. (b) Macroscopic score. (c) Toluidine Blue staining of rat knee joints and their sagittal section (left). Boxed regions represent higher magnification. Quantitative real‐time (qRT)‐PCR analysis of FBXO21, COL2A1, and MMP13, with β‐actin as the endogenous control, in (d) rat knee cartilage and (f) rat chondrocytes. 2 W: MIA‐2W; 20: 20 ng/ml of interleukin (IL)‐1β. Immunoblotting results of FBXO21, COL2A1, Aggrecan, MMP3, MMP13, LC3, Beclin1, Bcl2, and Bax, with β‐actin as the endogenous control, in the (e) knee cartilage of SD rats and (g) rat chondrocytes. (h) mRFP‐GFP‐LC3 adenovirus double label and (i) the quantification of GFP and mRFP dots per cell in the chondrocytes. mRFP (red) represents autolysosomes (ALs), Merge (yellow) represents autophagosomes (APs). (j) Ultrastructural features of APs in chondrocytes observed using transmission electron microscopy. Black box represents higher magnification. Blue arrows show APs. (k) Flow cytometry analysis. Data are presented as the mean ± SD; ns: not significant, *p < 0.05, **p < 0.01, ***p < 0.001, ****p < 0.0001

To further identify the role of FBXO21 in OA treatment, SD rats were preinjected with either overexpression‐FBXO21 (OE‐FBXO21) or overexpression‐NC (OE‐NC) adenovirus as described in Figure 3a. Gross imaging, plain radiograph, MRI, and macroscopic score of gross imaging showed significantly increased surface roughening, fibrillation, fissures, and erosions down to subchondral bone after FBXO21 overexpression (Figure 4a,b). Based on the staining results, rat knee joints showed significantly increased cartilage degradation, lower cartilage thickness, and higher OARSI grade (Figure 4c, Figure S4a,b). Chondrocytes of SD rats with OA (stimulated with 20 ng/ml of IL‐1β) were transfected with FBXO21 overexpression adenovirus. Overexpression efficiency of FBXO21 and corresponding phenotype markers in vivo and in vitro were confirmed by qRT‐PCR and immunoblotting. We found that FBXO21 expression was increased, whereas the levels of protective markers, including COL2A1, aggrecan, LC3 II/I, Beclin1, and Bcl2/Bax, were downregulated in vivo, except for those of the COL2A1 mRNA and aggrecan protein. Among the destructive markers, the expression of MMP3 was upregulated in vivo, but that of MMP13 mRNA was not (Figure 4d–g, Figure S4c,d). mRFP‐GFP‐LC3 adenovirus double label and TEM revealed that ALs and APs were decreased (Figure 4h–j, Figure S4e), and apoptosis was upregulated after FBXO21 overexpression (Figure 4k, Figure S4f). Thus, these results suggest that FBXO21 overexpression promotes OA‐related degeneration in MIA‐treated rats and IL‐1β‐treated rat chondrocytes.

### 2.5 FBXO21 enhances OA‐related degeneration in IL‐1β‐treated rat chondrocytes by inhibiting autophagy via interacting with and phosphorylating ERK

**FIGURE 5:**
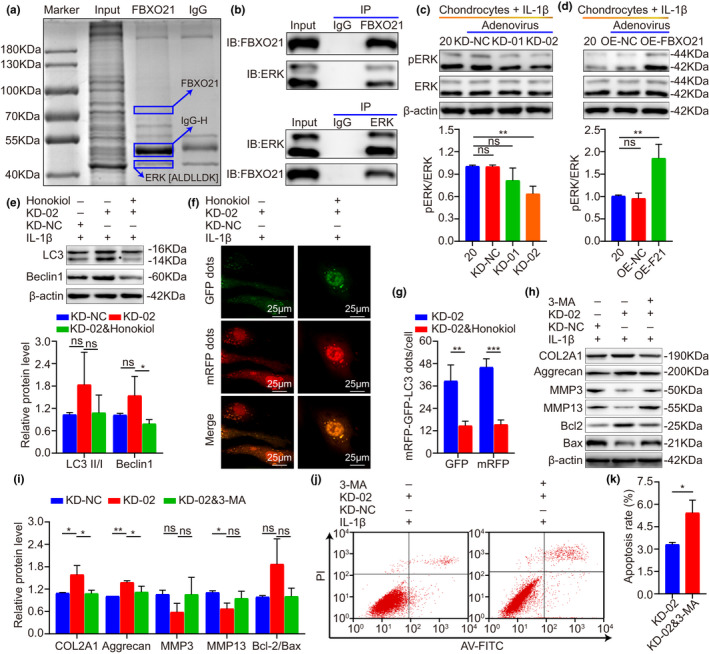
FBXO21 enhances OA‐related degeneration by interacting with and phosphorylating ERK, which inhibits autophagy in IL‐1β‐treated rat chondrocytes. (a) Immunoprecipitation (IP) with anti‐FBXO21 antibody of chondrocytes. The FBXO21‐IP protein complex was separated using sodium dodecyl sulfate‐polyacrylamide gel electrophoresis (SDS–PAGE), stained with Coomassie Brilliant Blue, digested by proteases, and quantified using LC–MS/MS. The identified peptide sequence of ERK is shown as (ALDLLDK). (b) FBXO21 (upper) and ERK (lower) proteins immunoprecipitated from chondrocytes with anti‐FBXO21 and anti‐ERK antibodies, respectively, were analyzed with immunoblotting. Immunoblotting results (upper) of pERK and ERK and their quantification (lower), with β‐actin as the endogenous control, after transfection with FBXO21 (c) knockdown and (d) overexpression adenovirus in the chondrocytes of SD rats. (e) Immunoblotting results (upper) of LC3 and Beclin1 and their quantification (lower), with β‐actin as the endogenous control, after treatment with FBXO21‐knockdown adenovirus‐02 (KD‐02) and the ERK activator Honokiol. (f) mRFP‐GFP‐LC3 adenovirus double label and the (g) quantification of GFP and mRFP dots per cell. mRFP (red) indicates autolysosomes; merge (yellow) indicates autophagosomes. (h) Immunoblotting and (i) quantification of COL2A1, Aggrecan, MMP3, MMP13, Bcl2, and Bax in chondrocytes treated with KD‐02 and the autophagy inhibitor 3‐methyladenine (3‐MA). (j) Flow cytometry analysis and (k) quantification. Data are presented as the mean ±SD; ns: not significant, *p < 0.05, **p < 0.01, ***p < 0.001

Then, we investigated the underlying mechanism of the FBXO21‐mediated enhanced cartilage degeneration of OA (stimulated with 20 ng/ml of IL‐1β). Initially, we performed immunoprecipitation (IP), Coomassie Brilliant Blue staining, and proteomic analysis (LC–MS/MS) to identify the FBXO21‐interacting proteins in chondrocytes of SD rats. Interestingly, the peptide sequence of the ERK protein was detected in the purified complex and there was no ERK protein in the 42‐kDa band of IgG binding protein (Table S3), suggesting that ERK binds to FBXO21 (Figure 5a). The interaction between ERK and FBXO21 was then confirmed in rat chondrocytes (Figure 5b). Next, we determined whether FBXO21 phosphorylates ERK in chondrocytes and found that FBXO21 markedly increased the levels of phosphorylated‐ERK (Figure 5c,d). Then, we performed immunoblotting and mRFP‐GFP‐LC3 adenovirus double label to identify whether FBXO21 regulates autophagy by phosphorylating ERK. In addition, the KD‐02‐mediated upregulation of ALs and APs were reversed after treatment with the ERK activator Honokiol (Figure 5d–g). Thus, we speculated that FBXO21 attenuates autophagy by phosphorylating ERK in chondrocytes. Furthermore, we examined in vitro by immunoblotting and flow cytometry analysis to determine whether FBXO21 regulates degeneration via autophagy. We observed that KD‐02‐mediated decreased apoptosis levels and that the upregulation of COL2A1 and Aggrecan were significantly reversed after treatment with the autophagy inhibitor 3‐methyladenine (3‐MA) (Figure 5h–k). We speculated that FBXO21 enhances degeneration by attenuating autophagy in chondrocytes. Taken together, these results suggest that FBXO21 enhances OA‐related degeneration in IL‐1β‐treated rat chondrocytes via interaction with and phosphorylation of ERK, resulting in the inhibition of autophagy.

### 2.6 JUNB accelerates OA‐related degeneration by regulating FBXO21 expression

**FIGURE 6:**
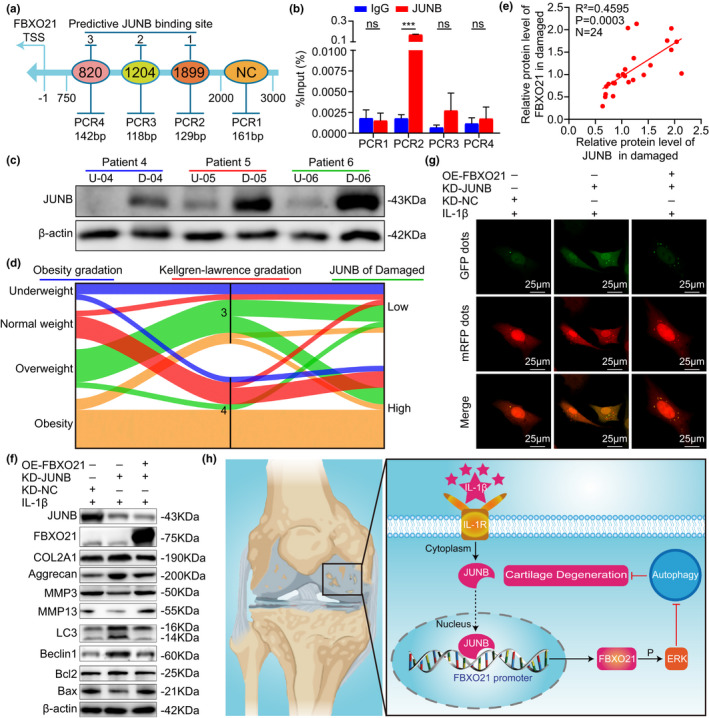
JUNB accelerates OA‐related degeneration by promoting FBXO21 expression. (a) Schematic of the predictive binding site of JUNB at the FBXO21 promoter region 2000‐bp upstream of the transcription start site (TSS) and the TSS, which is designated as −1. 2,000–3,000 bp was used as the negative control (NC). (b) Immunoprecipitated DNA using chromatin immunoprecipitation (ChIP) assay was quantified by PCR in SW1353 cells, with normal rabbit IgG as the NC. (c) Immunoblotting results of JUNB in undamaged (U) and damaged (D) areas in the cartilage of patients with knee osteoarthritis (OA), with β‐actin as the endogenous control. U‐04: undamaged (U) area of patient‐04; D‐04: damaged (D) area of patient‐04. (d) Relationship between obesity gradation, Kellgren–Lawrence gradation and JUNB expression in the damaged area represented by a Sankey diagram. (e) Linear regression analysis of the expression of JUNB and FBXO21 in the damaged area in patients with knee OA (*n* = 24). (f) Immunoblotting results of JUNB, FBXO21, COL2A1, Aggrecan, MMP3, MMP13, LC3, Beclin1, Bcl2, and Bax in SW1353 cells transfected with FBXO21‐pEX3 overexpression plasmid (OE‐FBXO21) and JUNB knockdown plasmid divided into KD‐shRNA‐NC (KD‐NC) and KD‐shRNA‐JUNB (KD‐JUNB) groups, with β‐actin as the endogenous control. (g) mRFP‐GFP‐LC3 adenovirus double label in SW1353 cells after JUNB knockdown and FBXO21 overexpression. mRFP (red) indicates autolysosomes; merge (yellow) indicates autophagosomes. (h) Schematic diagram showing the proposed mechanism of accelerated cartilage degeneration by the JUNB/FBXO21/ERK axis via regulating autophagy during OA. Data are presented as the mean ± SD; ns: not significant, ***p < 0.001

To verify the reason why expression of FBXO21 is upregulated during the development of OA (stimulated with 20 ng/ml of IL‐1β), first, we used the JASPAR database to predict the potential TFs of FBXO21 (*H. sapiens*) (Figure S5a) and found that JUNB is one of the predictive TFs that had been previously reported to be upregulated in OA (Rhee et al., 2017). To investigate whether JUNB directly targets the promoter region of FBXO21, we performed chromatin immunoprecipitation (ChIP) experiment and PCR. ChIP results found a direct target between JUNB and the FBXO21 promoter at the predictive binding site 1 (96.43‐fold) (Figure 6a,b). To further identify the clinical significance of JUNB expression and the correlation between JUNB and FBXO21 in OA, we tested the relative protein levels of JUNB by immunoblotting. Immunoblotting results indicated that JUNB was upregulated in the damaged cartilage of patients with knee OA (Figure 6c, Figure S5b) and that JUNB was positively correlated with FBXO21 level in damaged cartilage (Figure 6e) and weakly correlated in undamaged cartilage (Figure S5d). The relationship between patient characteristics, namely, age, gender, obesity gradation, and Kellgren–Lawrence gradation and the JUNB expression in the damaged area of the cartilage are presented in the Sankey diagram (Figure 6d, Figure S5c). Next, we performed immunoblotting, mRFP‐GFP‐LC3 adenovirus double label, and flow cytometry analysis to identify whether JUNB regulates degeneration by transcriptional activation of FBXO21. We observed that KD‐JUNB‐mediated decreased MMP3, MMP13, and apoptosis levels and increased COL2A1, aggrecan, and autophagy levels significantly reversed after overexpression of FBXO21 (Figure 6f,g, Figures S5e–h and S6a,b). Thus, these results indicate that JUNB accelerates OA‐related degeneration by activating the expression of FBXO21 in SW1353 cells and rat chondrocytes.

Hence, based on our findings, we proposed a mechanism involving the JUNB/FBXO21/ERK axis that participates in the progression of OA cartilage degeneration by regulating autophagy (Figure 6h).

## 3 DISCUSSION

OA is extremely hard to cure owing to its complex pathogenesis involving the cross‐regulation of spatial networks having multiple pathways and phenotypes such as ERK pathway, autophagy, apoptosis, anabolism, and catabolism (Glyn‐Jones et al., 2015; Ji et al., 2019; Kim et al., 2014; Loeser et al., 2016; Lotz & Caramés, 2011; Rahmati et al., 2017; Zhang et al., 2017). Thus, its pathogenesis remains to be fully elucidated. Considering that the JNK and p38 pathways have been reported to be activated by FBXO21 (Yu et al., 2016) and that they promote OA progression (Zhou et al., 2019), we hypothesized that FBXO21 is dysregulated in OA and plays a crucial role in its pathogenesis. Our findings verified that FBXO21 was indeed highly expressed in rat chondrocytes, rat cartilage with OA, and cartilage of patients with OA. Thus, it can be used to predict the severity of OA, especially in terms of Kellgren–Lawrence gradation. As patients with end‐stage OA who can undertake total knee arthroplasty are mostly middle‐aged and elderly, and the 24 patients with knee OA we could collect were from 57 to 82 years old. Therefore, in this study, we only set up two groups for 24 patients with OA: the relatively younger (<65 years old) and older group (≥65 years old). And FBXO21 expression was not significantly different between the two groups. It proved that FBXO21 expression was not necessarily aging associated between 57 and 82 years old. In the rat data, the 12‐month‐old rats were used to simulate the relatively younger group (<65 years old), and the 18‐month‐old rats were used to simulate the older group (≥65 years old). And FBXO21 expression was not significantly different between 12‐month‐old and 18‐month‐old rats, which was consistent with the conclusions of human data. In vivo and in vitro experiments revealed that FBXO21 knockdown suppressed OA‐related cartilage degeneration as evidenced by activated autophagy, upregulated anabolism, alleviated apoptosis, and downregulated catabolism. In contrast, its overexpression promoted OA‐related cartilage degeneration. However, the complex mechanisms involved in the regulation of cartilage degeneration by FBXO21 require further exploration.

To elucidate the detailed molecular mechanism involved in the regulation of cartilage degeneration by FBXO21, we detected that ERK is one of the binding proteins of FBXO21 by mass spectrometry (MS) and co‐immunoprecipitation (co‐IP) assays. ERK belongs to the same pathway as that of JNK and p38, which could be activated by FBXO21 (Yu et al., 2016). Next, we confirmed that FBXO21 knockdown impairs the ERK pathway, whereas its overexpression activates the said pathway. Thus, we hypothesized that FBXO21 regulates cartilage degeneration via the ERK pathway. However, ERK mainly affects cell proliferation, autophagy (Corcelle et al., 2007), and apoptosis but has little effect on cartilage matrix metabolism (Latourte et al., 2017; Prasadam et al., 2010, 2013). We then speculated that FBXO21 first regulates autophagy by ERK pathway. Consistent with this, the autophagy activated by FBXO21 knockdown was reversed by the ERK activator Honokiol, suggesting that FBXO21 inhibits autophagy by interacting with and phosphorylating ERK. ERK is one of the novel binding partners of FBXO21 detected in the LC‐MS, there are still numerous novel FBXO21‐binding partners to be verified like catenin beta‐1. FBXO21 may regulate OA‐related degeneration by affecting catenin beta‐1.

Although we confirmed the possible mechanism of how FBXO21 regulates autophagy through the ERK pathway, the mechanisms of FBXO21 in regulating apoptosis and cartilage matrix metabolism remain to be elucidated. Previous reports have implied that ERK has little effect on cartilage matrix metabolism (Latourte et al., 2017; Prasadam et al., 2010, 2013); on the contrary, autophagy can upregulate anabolism, alleviate apoptosis and downregulate catabolism in the late stage of OA (Kang & Elledge, 2016; Li et al., 2016). Thus, we propose that FBXO21 activates apoptosis and cartilage matrix metabolism by autophagy, which is regulated by the ERK pathway. Consistent with this, the observed upregulated anabolism, alleviated apoptosis, and downregulated catabolism following FBXO21 knockdown were reversed by the autophagy inhibitor 3‐MA. Hence, FBXO21 is suggested to alleviate anabolism and enhance apoptosis and catabolism by inhibiting autophagy.

To determine why FBXO21 is upregulated in OA, we predicted a multitude of TFs in the promoter region of FBXO21 (*H. sapiens*) using the JASPAR database (Fornes et al., 2020). We selected JUNB as the predicted upstream TF of FBXO21, because it has been reported to bind to the promoter region of MMP13 and promote the expression of MMP13 in OA (Rhee et al., 2017). We first showed that JUNB binds to the promoter region of FBXO21. Then, we found that JUNB was upregulated and positively correlated with FBXO21 expression in the damaged cartilage of patients with knee OA but not in undamaged areas. Thus, we speculated that JUNB regulates cartilage degeneration by targeting FBXO21. Consistent with this, its knockdown effects, that is, activated autophagy, upregulated anabolism, alleviated apoptosis, and downregulated catabolism and expression of FBXO21, were reversed by the overexpression of FBXO21 in SW1353 cells and rat chondrocytes. Hence, these findings support that JUNB inhibits autophagy and anabolism and enhances apoptosis and catabolism by directly targeting the FBXO21 promoter.

However, our study has several limitations. First, we only studied young and male SD rats. The chondro‐destructive effects of FBXO21 should be confirmed in female rats, obese rats, and aging‐associated OA models. Second, we only employed noninvasive examination, including plain radiograph and MRI, which cannot be used for quantification. To further identify the function of FBXO21 in OA, we recommend the use of micro‐computed tomography and MRI with high resolution. Third, we observed high levels of FBXO21 in the nuclei of chondrocytes, and its specific role in the nucleus should be explored. Lastly, we used the SW1353 cell line and rat chondrocytes for our OA studies. Studies suggest that SW1353 cells have very little similarity with human articular chondrocytes, to confirm that JUNB indeed accelerates degeneration by activating FBXO21 expression, the relationship between JUNB and FBXO21 should be investigated using human chondrocytes.

In conclusion, we demonstrated that FBXO21 was upregulated in chondrocytes and the cartilage of rats and patients with OA. Importantly, we propose that the JUNB‐FBXO21‐ERK axis promotes OA cartilage degeneration by inhibiting autophagy (Figure 6h), thus providing a novel targeted therapy for OA.

## 4 EXPERIMENTAL PROCEDURES

### 4.1 Human OA cartilage and clinicopathological features

This study has been approved by the Ethics Committee of Shengjing Hospital of China Medical University (Shenyang, China; approval number: 2019PS629K). Informed consent was obtained from all patients. The cartilage tissues and clinicopathological features of 24 patients with knee OA who underwent total knee arthroplasty were collected. Articular cartilage samples were divided into two groups according to the International Cartilage Repair Society (ICRS) grade (van den Borne et al., 2007). Undamaged area with ICRS = 0 was grouped together, whereas those with ICRS = 1–4 was in the damaged area.

### 4.2 Animals

The study protocol was approved by the Animal Care and Use Committee of the China Medical University (Shenyang, China; approval number: 2017PS237K), and all animal procedures involving SD rats were performed in complete compliance with Health Guide of Laboratory Animals for the Care and Use from the National Institutes (National Research Council Committee for the Update of the Guide for the & Use of Laboratory, 2011) and ARRIVE guidelines (Kilkenny et al., 2010). All efforts to minimize pain and suffering in SD rats were used.

We established two OA models consisting of Aging and MIA (Aike Reagent) induced to mimic OA in SD rats. The aging model was divided into four groups according to age (*n* = 6 per group): 1 month old (1 M), 6 months old (6 M), 12 months old (12 M), and 18 months old (18 M). The MIA model (induced by the injection of 40 µl saline with or without 0.2 mg MIA into the intra‐articular cavity of 8‐week‐old SD rats after anesthetization with isoflurane as previously described) (Lu et al., 2020; Udo et al., 2016; H. Zhang et al., 2019) was also divided into four groups (*n* = 6 per group) according to MIA treatment time: saline control (Ctrl), MIA treatment for 1 week (1 W), MIA treatment for 2 weeks (2 W), and MIA treatment for 3 weeks (3 W).

To identify the role of FBXO21, recombinant adenovirus with *FBXO21* knockdown or overexpression was first injected into the intra‐articular cavity of 7‐week‐old SD rats three times weekly (30 µl of 10^10^ PFU/ml; Hanbio, Shanghai, China) before the injection of MIA for 2 weeks (2 W) in 8 weeks old. Thus, rats with *FBXO21* knockdown were divided into three groups (*n* = 6 per group): 2 W + sh‐NC (KD‐NC), 2 W + sh‐FBX021‐01 (KD‐01), and 2 W + sh‐FBX021‐02 (KD‐02). Rats with *FBXO21* overexpression were divided into two groups (*n* = 6 per group): 2 W + Ad‐NC (OE‐NC) and 2 W + Ad‐FBXO21‐3xflag‐pAdTrack‐CMV (OE‐FBXO21).

Overall, 78 male SD rats (200 ± 10 g, 6 weeks old) provided by HFK Bioscience Cooperation were divided into thirteen groups (*n* = 6 per group). All SD rats were maintained in a specific pathogen‐free (SPF) environment kept at a constant temperature of 22°C and 70% humidity and fed with a standard diet.

### 4.3 Plain radiography

After the end of the experimental cycle, SD rats were anesthetized by intraperitoneal injection of pentobarbital sodium (40 mg/kg). Plain radiographs of the knee joints were photographed using an X‐ray machine (MS FX PRO; BRUKER) operated by a technician, in which the bilateral ankles of SD rats were fixed on the pallet as previously described (Zhang et al., 2019). The images were evaluated based on the narrowing of joint space and osteophytes formation (Kohn et al., 2016).

### 4.4 Magnetic resonance imaging

MRI of the knee joints was performed using a Ingenia3.0 T MRI system (Philips) operated by a technician, in which bilateral ankles of SD rats were fixed on the pallet. Briefly, knee joints were surveyed twice for accuracy of location after placing the special coil. Then, sagittal and coronal sections were scanned with the 3D_WATSc program. MRI scan evaluation was based on cartilage loss, osteophytes formation, and subchondral sclerosis (Hunter et al., 2011).

### 4.5 Gross imaging

SD rats were euthanized by cervical dislocation following MRI. The whole left joints were placed in 4% paraformaldehyde for histological analysis, whereas the right joints were dissected on ice to fully expose the cartilage of the femur and tibia. Gross imaging of the knee joint cartilage was photographed using a stereomicroscope (XTL‐165‐CB; Phenix). Macroscopic scoring was based on surface roughening, fibrillation, fissures, and erosions down to subchondral bone (Gerwin et al., 2010) and was performed by two researchers in a blinded manner.

The knee joint cartilages of SD rats were isolated from tibial plateaus and femoral condyles using a scalpel, washed with phosphate‐buffered saline (PBS), and stored at −80°C for subsequent experiments.

### 4.6 Culture of primary articular chondrocytes and SW1353 cell line

Primary articular chondrocytes were harvested from the hip and knee articular cartilage of 3‐week‐old male SD rats (80 ± 10 g; SPF). After the cartilage was digested with pronase K (1.5 mg/ml, Roche) for 60 min at 37°C and collagenase D (1.2 mg/ml; Roche) for 60 min at 37°C in that order, the primary chondrocytes were seeded in culture flasks (25 cm^2^) with 1% streptomycin, 1% penicillin (Hyclone), 10% fetal bovine serum (FBS) (Bioind, China), and Dulbecco's modified Eagle's medium (DMEM) (Hyclone) in 5% CO_2_ atmosphere at 37 °C. Upon reaching 80% confluence, the primary chondrocytes were divided in a 1:3 ratio and stained with Alcian Blue (Solarbio), Safranin O (Solarbio), or Toluidine Blue (Solarbio) according to the manufacturer's instructions. Immunohistochemistry (IHC) staining for collagen II (1:200 dilution; E‐AB‐70208; Elabscience) was also performed.

To ensure the phenotype of primary chondrocytes and accuracy of follow‐up experiments, only 2–3 passages were used. The human chondrosarcoma cell line SW1353 was maintained in 1% streptomycin, 1% penicillin (Hyclone), 10% FBS (Bioind), and DMEM (Hyclone) in 5% CO_2_ at 37°C. To induce OA in vitro, rat primary chondrocytes were treated with recombinant rat IL‐1β for 12 h (0, 5, 10, or 20 ng/ml; Beyotime, China), recombinant rat TNFα for 12 h (0, 5, 10, or 20 ng/ml; Beyotime), and LPS for 6 h (0, 0.5, 1.0, or 2.0 μg/ml; Sigma, USA). Meanwhile, SW1353 cells were treated with recombinant human IL‐1β for 12 h (0, 5, 10, or 20 ng/ml; Beyotime), recombinant human TNFα for 12 h (0, 5, 10, or 20 ng/ml; Beyotime) and LPS for 6 h (0, 0.5, 1.0, or 2.0 μg/ml; Sigma) (Kapoor et al., 2011).

To determine the role of FBXO21 in OA treatment, primary chondrocytes were transfected with recombinant adenovirus with *FBXO21* knockdown or overexpression (multiplicity of infection [MOI] = 50, polybrene = 3 μg/ml; Hanbio). Then, the primary chondrocytes were harvested after stimulation with IL‐1β (20 ng/ml) for 12 h. Thus, knockdown of FBXO21 was divided into three groups: IL‐1β + sh‐NC (KD‐NC), IL‐1β + sh‐FBX021‐01 (KD‐01), and IL‐1β + sh‐FBX021‐02 (KD‐02). Overexpression of FBXO21 was divided into two groups: IL‐1β + Ad‐NC (OE‐NC) and IL‐1β + Ad‐FBXO21‐3xflag‐pAdTrack‐CMV (OE‐FBXO21).

To investigate whether FBXO21 regulates autophagy by phosphorylating ERK in vitro, chondrocytes were transfected with the KD‐02 adenovirus and harvested after simultaneous stimulation with IL‐1β (20 ng/ml) and the ERK activator Honokiol (5 μM; MCE) for 12 h. To explore whether FBXO21 regulates OA‐related degeneration by autophagy, chondrocytes were transfected with the KD‐02 adenovirus and harvested after simultaneous stimulation with IL‐1β (20 ng/ml) and the autophagy inhibitor 3‐methyladenine (3‐MA; 5 mM; MCE) for 12 h.

To identify whether JUNB regulates cartilage degeneration via the transcriptional activation of FBXO21, the SW1353 cell line was transfected with the knockdown plasmid of NC (KD‐NC) and JUNB (KD‐JUNB) (GenePharma) and the overexpression plasmid of FBXO21‐pEX3 (OE‐FBXO21) (GenePharma) by lipo3000 (Invitrogen) according to the manufacturer's instructions. We used the human chondrocyte SW1353 cell line because the transcription factors of FBXO21 predicted by the JASPAR database is found in *H. sapiens*. Rat chondrocytes were transfected with *JUNB* knockdown or *FBXO21* overexpression recombinant adenovirus (MOI = 50, polybrene = 3 μg/ml; Hanbio) and harvested after stimulation with IL‐1β (20 ng/ml) for 12 h.

### 4.7 Transmission electron microscopy

Primary chondrocytes were harvested after treating and fixing in 2.5% glutaraldehyde (pH 7.4) overnight and 1% osmium tetroxide for 2 h at 4°C. TEM (Hitachi) was performed after dehydration, infiltration, imbedding, and sectioning.

### 4.8 Autophagy flux detection

Primary chondrocytes were seeded on cell climbing slides and transfected with the mRFP‐GFP‐LC3 adenovirus (MOI = 50; polybrene = 3 μg/ml; Hanbio) before or meanwhile. Chondrocytes were treated as described in 4.6 and fixed in 4% paraformaldehyde for 30 min at room temperature and imaged using a two‐photon fluorescence microscope (Zeiss).

### 4.9 Immunoprecipitation, Coomassie Brilliant Blue staining, and Proteomics of mass spectrometry (MS)

Total protein was incubated with 1 μg of normal rabbit IgG (A7016; Beyotime) as control or 1 μg of anti‐FBXO21 antibody (Ab179818; Abcam) overnight at 4°C after the extraction of primary chondrocytes using combination of RIPA buffer for immunoprecipitation (IP; P0013; Beyotime), Phenylmethanesulfonyl fluoride (PMSF; Beyotime), and phosphatase inhibitors (Beyotime) at a ratio of 100:1:1. The mixtures of protein and antibody were subsequently incubated with 40 μl of pre‐washed protein A/G PLUS‐Agarose beads (sc‐2003; Santa Cruz) in gentle rotation for 4 h at 4°C. Next, the immunoprecipitates were separated by sodium dodecyl sulfate‐polyacrylamide gel electrophoresis (SDS‐PAGE) on 10% agarose gel and stained by Coomassie Brilliant Blue (P0017F; Beyotime). Mass spectrometry (Novogene) and immunoblotting were performed (Supporting Information).

### 4.10 Transcription factor prediction and chromatin immunoprecipitation assay

We used the NCBI gene database to search for the promoter region of FBXO21 and the JASPAR 2018 TFBS track of the University of California, Santa Cruz (UCSC) Genome Browser (Fornes et al., 2020) for TFs. According to the predicted score of TFs (Figure S5a) and previous associations with OA, we focused on JUNB and predicted its binding sites in the promoter region of FBXO21 using the JASPAR database.

Cellular lysates were harvested from SW1353 cells, and ChIP assay was conducted using an IP Kit of SimpleChIP Enzymatic Chromatin (9002S; CST) according to the manufacturer's protocol. Briefly, SW1353 cells stimulated with IL‐1β were fixed in 1% formaldehyde at room temperature for 10 min to cross link DNA and protein. Then, they were treated with Buffer A, Buffer B, Micrococcal Nuclease (10011; CST), and ChIP buffer in this sequence to extract fragmented chromatins. They were incubated overnight with normal rabbit IgG (2729; CST) as control or anti‐JUNB antibody (C37F9; CST) at 4°C. The mixtures of protein–DNA and antibody were subsequently incubated with Protein G Agarose Beads of ChIP‐Grade (9007; CST) at 4°C for 3 h with shaking. After washing three times with low and once with high‐salt, the protein‐DNA crosslinks of the immunoprecipitates were uncoupled by reaction with proteinase K for 2 h, and DNA was purified with DNA purification columns. Purified DNA was subjected to qRT‐PCR (Supporting information) with the primers provided by Sangon (China; Table S4).

### 4.11 Statistical analysis

All experiments were conducted at least three times, and data were presented as the mean ± SD. Data analysis was conducted using SPSS v24.0 (SPSS Inc.) or GraphPad Prism v8 (GraphPad Inc.). Normality and homogeneity were evaluated using Shapiro–Wilk and Levene tests, respectively. For values with normal distribution, parametric analysis included paired *t* test (two groups), unpaired *t* test (two groups), Welch's *t* test (two groups, unequal variances), and one‐way analysis of variance (ANOVA) (multiple groups). For values with non‐normal distribution, non‐parametric analysis included Mann–Whitney test (two groups) and Kruskal–Wallis test (multiple groups). Simple linear regression was used to evaluate the relationship between JUNB and FBXO21. Chi‐square test and Spearman correlation analysis were used to evaluate the relationship between FBXO21 expression and the baseline characteristics of patients with OA. *p* < 0.05 was considered significant. *R* ^2^ < 0.16 was considered low linear correlation; 0.16 ≤ *R* ^2^ < 0.49 significant correlation; and 0.49 ≤ *R* ^2^ < 1, high linear correlation.

## CONFLICT OF INTEREST

None declared.

## AUTHOR CONTRIBUTIONS

ZML and LHB designed the study. MH and ZYW collected clinical data and samples. ZML and JNM performed the in vivo and in vitro experiments. ZML, TZ, and XYF analyzed the data and prepared the figures. ZML wrote the manuscript. LHB revised the manuscript. All authors have read and approved the final version of the manuscript for publication.

## Supporting information

Fig S1

Fig S2

Fig S3

Fig S4

Fig S5

Fig S6

Supplementary Material

## Data Availability

Data that support the findings of this research are included in the published article and its supporting information.
