# Supplementary figures and images for: JUNB‐FBXO21‐ERK axis promotes cartilage degeneration in osteoarthritis by inhibiting autophagy
Source: Aging Cell. 2021 Jan 15;20(2):e13306. doi: 10.1111/acel.13306 (PMC7884044; doi:10.1111/acel.13306)

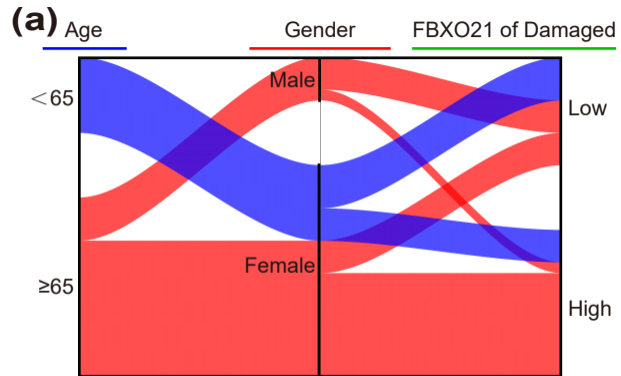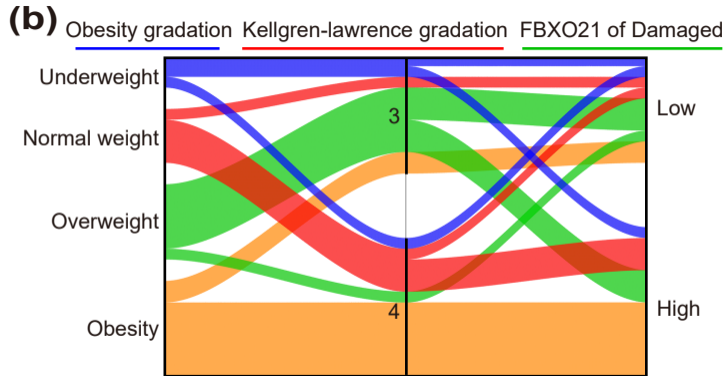

Supplement: Supplementary file 1 — Fig S1 [file ACEL-20-e13306-s001.pdf]

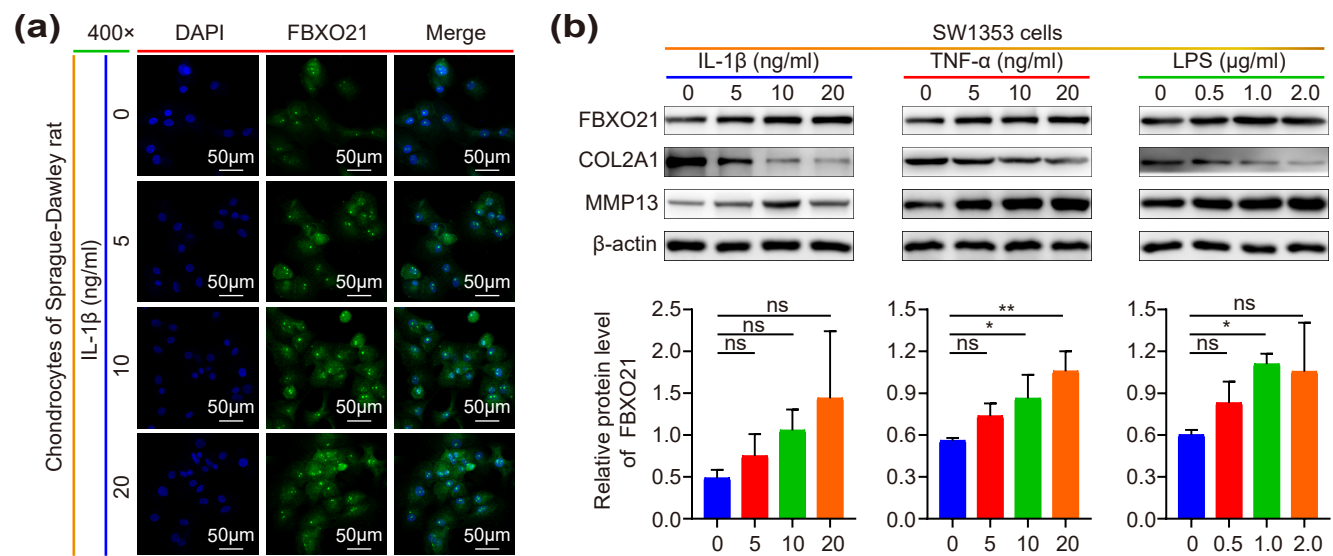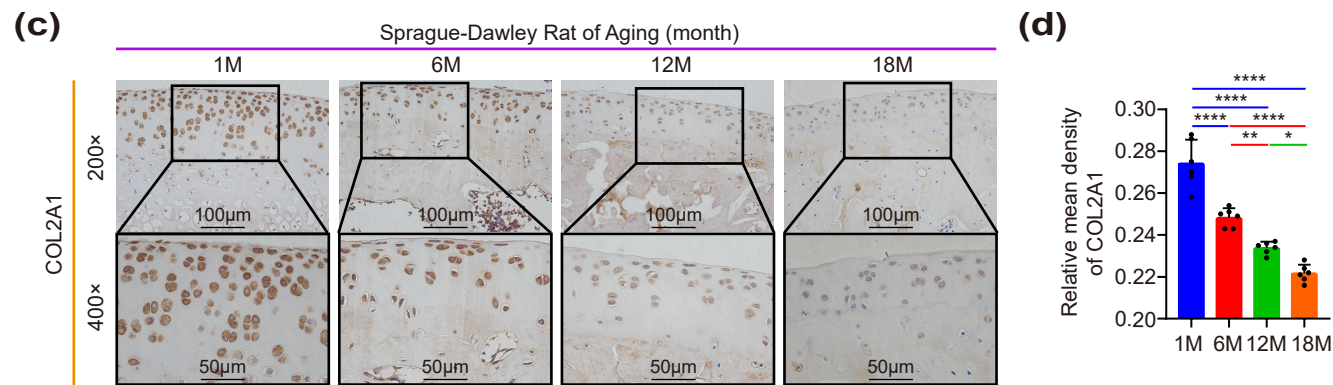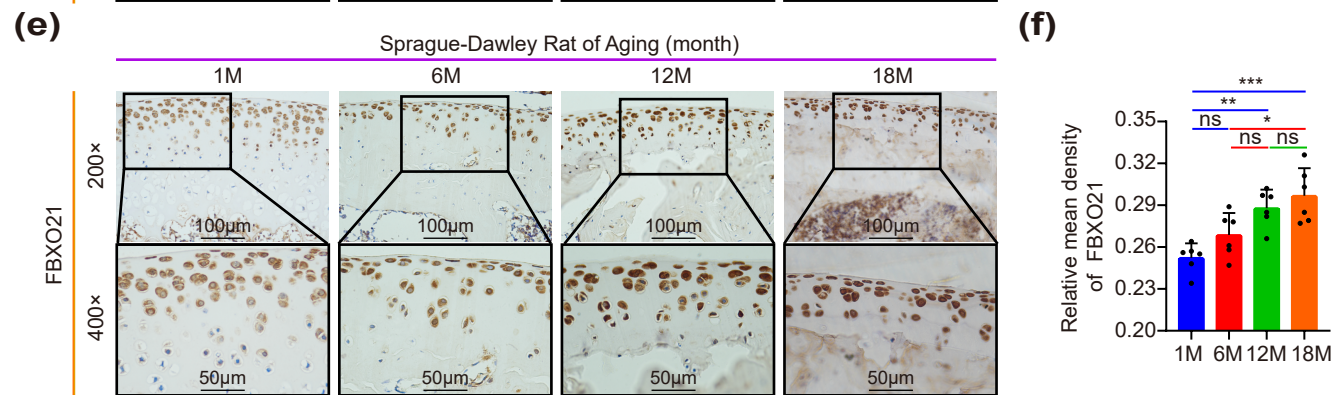

Supplement: Supplementary file 2 — Fig S2 [file ACEL-20-e13306-s002.pdf]

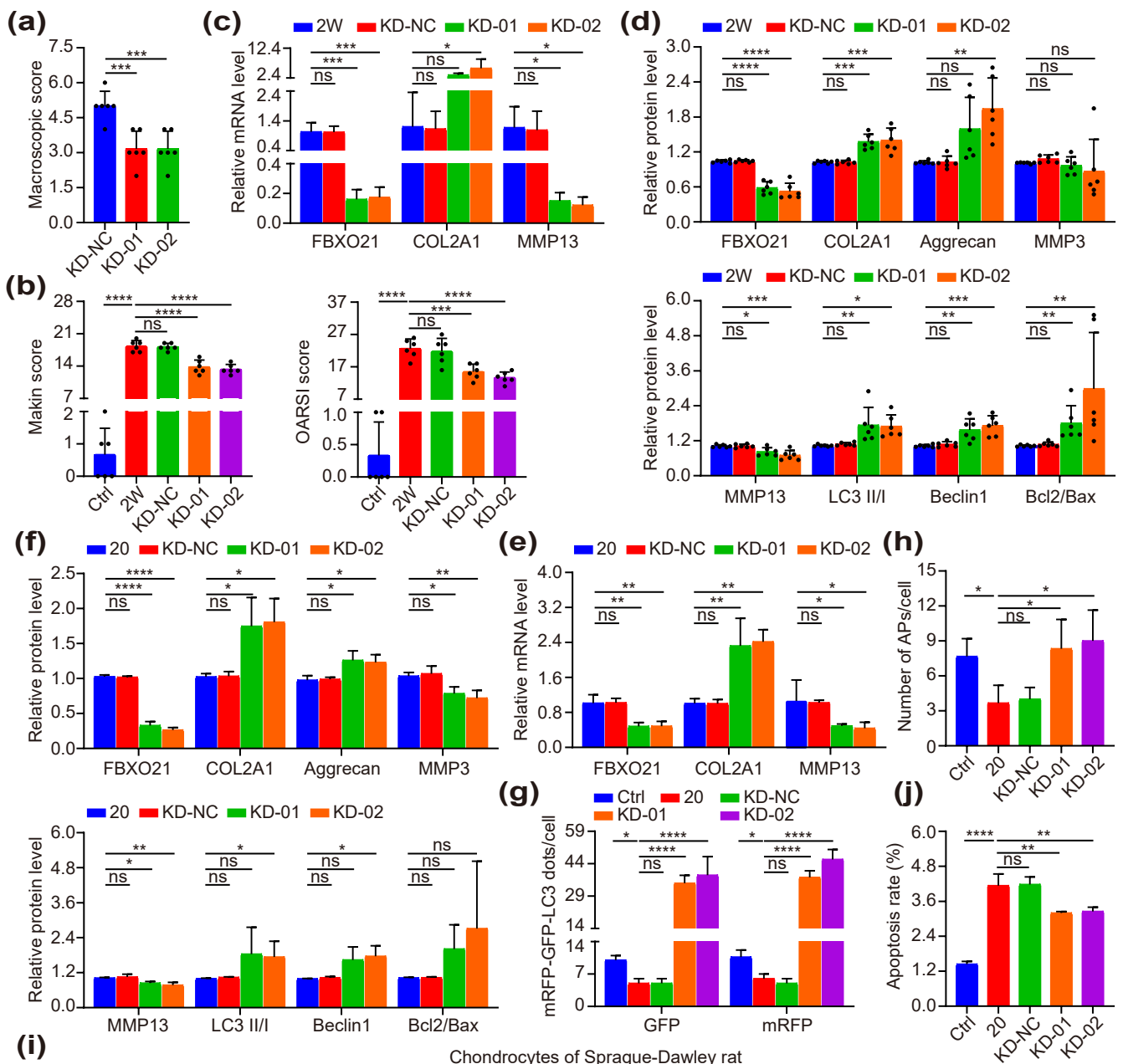

Chondrocytes of Sprague-Dawley rat

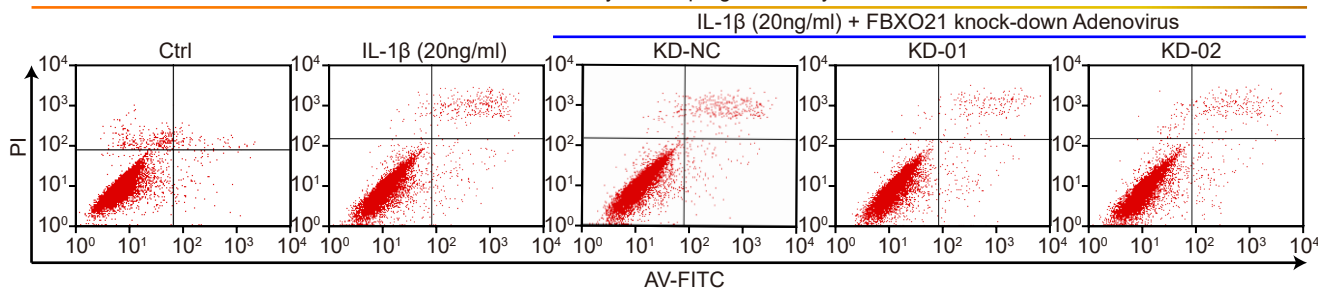

Supplement: Supplementary file 3 — Fig S3 [file ACEL-20-e13306-s003.pdf]

**(a)**

Sprague-Dawley Rat

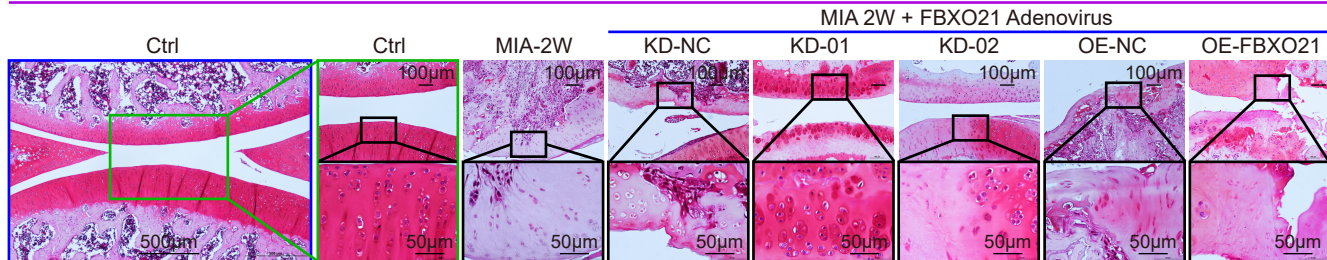**(b)**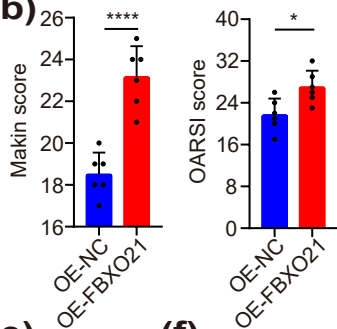**(c)**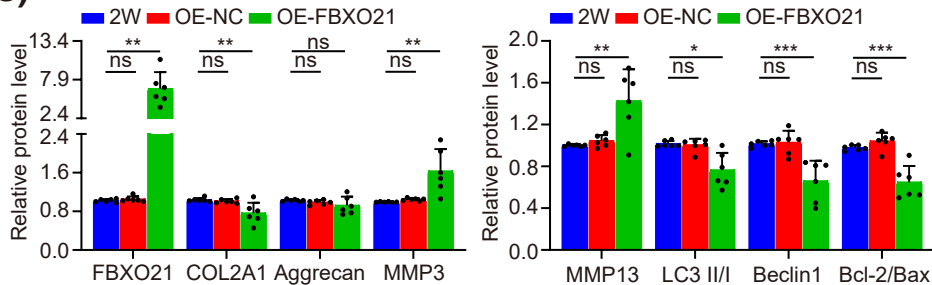**(e)**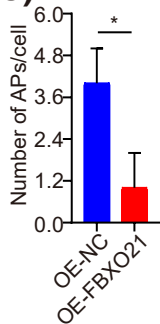**(f)**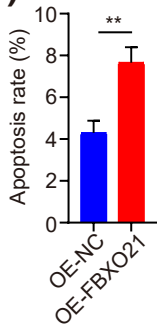**(d)**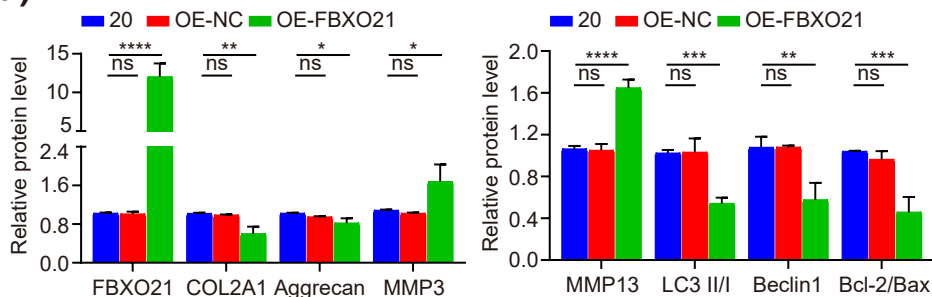

Supplement: Supplementary file 4 — Fig S4 [file ACEL-20-e13306-s004.pdf]

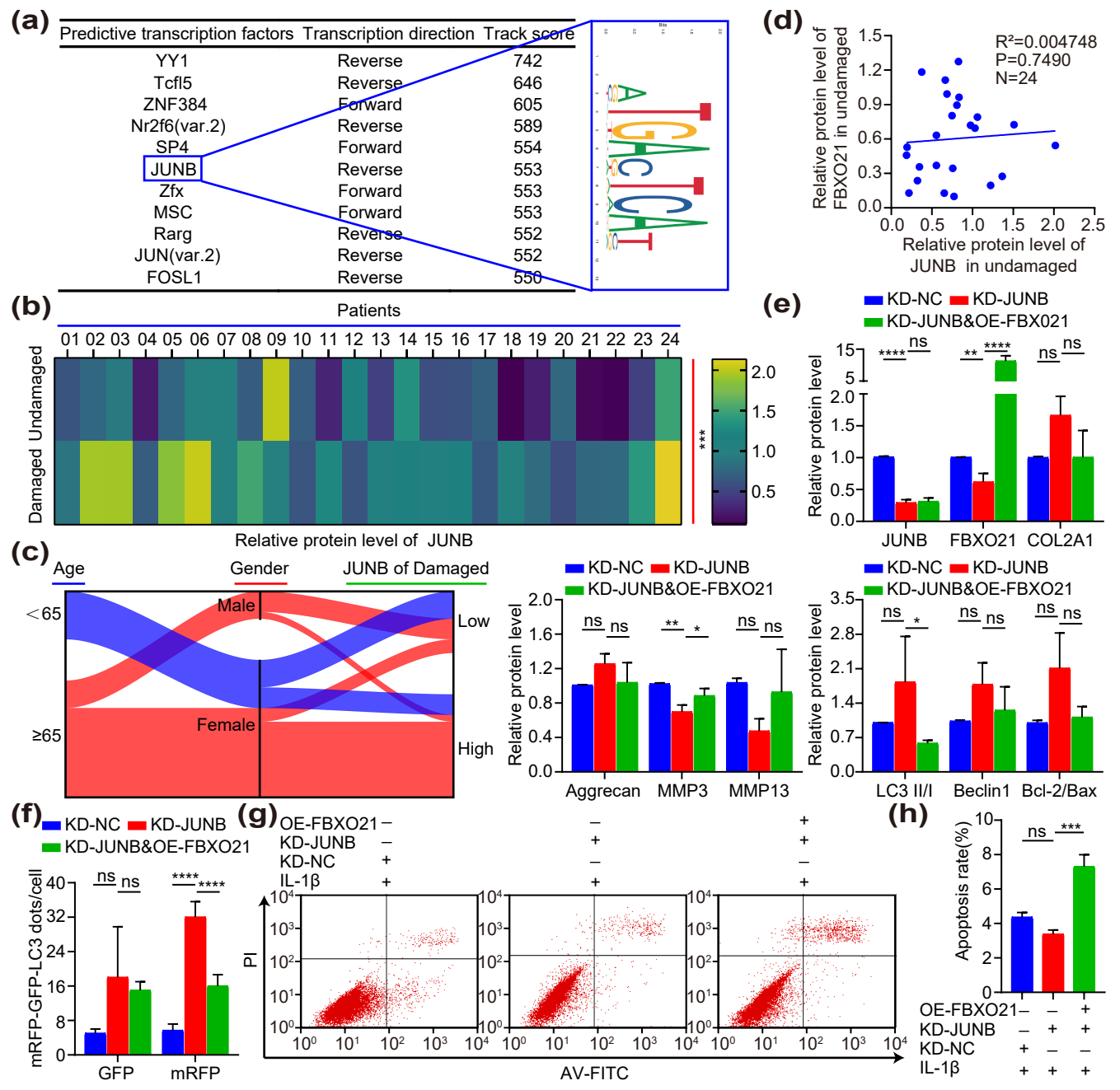

Supplement: Supplementary file 5 — Fig S5 [file ACEL-20-e13306-s005.pdf]

**(a)**

|              |   |   |   |
|--------------|---|---|---|
| OE-FBXO21    | - | - | + |
| KD-JUNB      | - | + | + |
| KD-NC        | + | - | - |
| IL-1 $\beta$ | + | + | + |

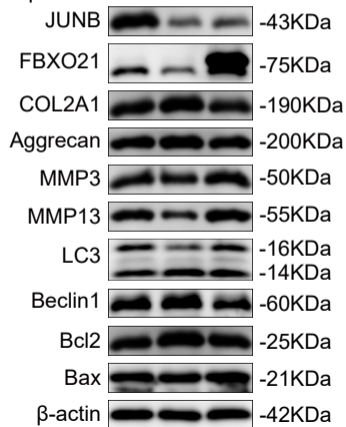**(b)**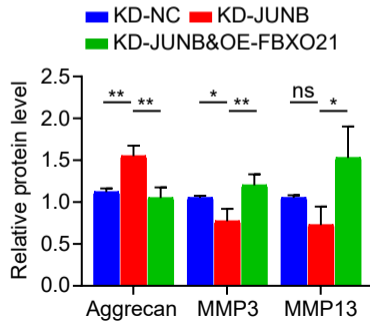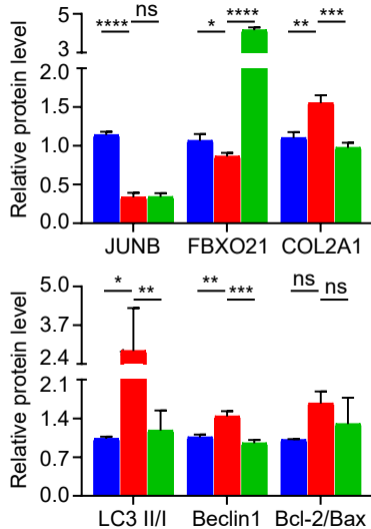

Supplement: Supplementary file 6 — Fig S6 [file ACEL-20-e13306-s006.pdf]
