# Supplementary material for: JUNB‐FBXO21‐ERK axis promotes cartilage degeneration in osteoarthritis by inhibiting autophagy
Source: Aging Cell. 2021 Jan 15;20(2):e13306. doi: 10.1111/acel.13306 (PMC7884044; doi:10.1111/acel.13306)
Supplement: Supplementary file 7 — Supplementary Material [file ACEL-20-e13306-s007.docx]

**1 | EXPERIMENTAL PROCEDURES**

**1.1 | knockdown sequence of rat-FBXO21**

KD-NC (siRNA sequence: TTCTCCGAACGTGTCACGTAA, Top strand of shRNA sequence: GATCCGTTCTCCGAACGTGTCACGTAATTCAAGAGATTACGTGACACGTTCGGAGAATTTTTTC, Bottom strand of shRNA sequence: AATTGAAAAAATTCTCCGAACGTGTCACGTAATCTCTTGAATTACGTGACACGTTCGGAGAACG).

KD-01 (siRNA sequence: AGGAAAGCTCTGACCTGGAAGTACT, Top strand of shRNA sequence: tcgagGAGGAAAGCTCTGACCTGGAAGTACTTTCAAGAGAAGTACTTCCAGGTCAGAGCTTTCCTTTTTTTa, Bottom strand of shRNA sequence: agcttAAAAAAAGGAAAGCTCTGACCTGGAAGTACTTCTCTTGAAAGTACTTCCAGGTCAGAGCTTTCCTCc).

KD-02 (siRNA sequence: TGGACGCCATCAACTATGTCCTGTA, Top strand of shRNA sequence: tcgagGTGGACGCCATCAACTATGTCCTGTATTCAAGAGATACAGGACATAGTTGATGGCGTCCATTTTTTa, Bottom strand of shRNA sequence: agcttAAAAAATGGACGCCATCAACTATGTCCTGTATCTCTTGAATACAGGACATAGTTGATGGCGTCCACc).

**1.2 | knockdown sequence of human-JUNB**

KD- NC (siRNA sequence: GTTCTCCGAACGTGTCACGT, Top strand of shRNA sequence: CACCGTTCTCCGAACGTGTCACGTTTCAAGAGAACGTGACACGTTCGGAGAACTTTTTTG, Bottom strand of shRNA sequence: GATCCAAAAAAGTTCTCCGAACGTGTCACGTTCTCTTGAAACGTGACACGTTCGGAGAAC).

KD- JUNB (siRNA sequence: GGAAGATTGTAGCCTCGTTCT, Top strand of shRNA sequence: CACCGGAAGATTGTAGCCTCGTTCTTTCAAGAGAAGAACGAGGCTACAATCTTCCTTTTTTG, Bottom strand of shRNA sequence: GATCCAAAAAAGGAAGATTGTAGCCTCGTTCTTCTCTTGAAAGAACGAGGCTACAATCTTCC).

**1.3 | knockdown sequence of rat-JUNB**

KD-NC (siRNA sequence: TTCTCCGAACGTGTCACGTAA, Top strand of shRNA sequence: GATCCGTTCTCCGAACGTGTCACGTAATTCAAGAGATTACGTGACACGTTCGGAGAATTTTTTC, Bottom strand of shRNA sequence: AATTGAAAAAATTCTCCGAACGTGTCACGTAATCTCTTGAATTACGTGACACGTTCGGAGAACG).

KD- JUNB (siRNA sequence: TCTGCTACTCCATCGGGCTCGTTAT, Top strand of shRNA sequence: tcgagGTCTGCTACTCCATCGGGCTCGTTATTTCAAGAGAATAACGAGCCCGATGGAGTAGCAGATTTTTTa, Bottom strand of shRNA sequence: agcttAAAAAATCTGCTACTCCATCGGGCTCGTTATTCTCTTGAAATAACGAGCCCGATGGAGTAGCAGACc).

**1.4 | Quantitative Reverse Transcription PCR**

Total RNA was harvested from knee joint cartilages and primary chondrocytes of SD rats using RNAiso Plus (Takara, Japan). PrimeScript RT reagent Kit (Takara) was used to reverse transcribe cDNA from 1 µg RNA after detection of RNA concentrations by micro ultraviolet spectrophotometer (NanoVne, GE Healthcare, UK). SYBR Premix Ex TaqTM II (Takara) was used to quantify expression of FBXO21, COL2A1 and MMP13 by polymerase chain reaction in LightCycler480 II (Roche, Switzerland). The fold change of relative mRNA expression was determined by 2−ΔΔCt with β-actin as control. The primers sequences were provided by Sangon (China) as shown in TABLE S2.

**1.5 | Immunoblotting**

Total protein was extracted from cartilage of patients with knee OA, knee joint cartilages of SD rats, primary chondrocytes and SW1353 cells using combination of RIPA buffer (Beyotime), PMSF (Beyotime) and phosphatase inhibitors (Beyotime) at ratio of 100:1:1. Proteins were separated by SDS‐PAGE on a 8-12% gel and wet transferred to polyvinylidene difluoride (PVDF) membranes (Millipore, USA) by constant flow. 5% non-fat milk used to block membranes for 2 h at room temperature. Next, membranes were incubated at 4°C overnight with primary antibodies as follows: anti-FBXO21 antibody (1:10000, Ab179818, Abcam), anti-COL2A1 antibody (1:1000, E-AB-70208, Elabscience), anti-Aggrecan antibody (1:1000, 13880-1-AP, Proteintech, USA), anti-MMP3 antibody (1:1000, E-AB-70298, Elabscience), anti-MMP13 antibody (1:1000, E-AB-70346, Elabscience), anti-LC3B antibody (1:2000, Ab192890, Abcam), anti-Beclin1 antibody (1:2000, Ab207612, Abcam), anti-Bcl2 antibody (1:1000, Ab32124, Abcam), anti-Bax antibody (1:3000, Ab32503, Abcam), anti- phospho-ERK antibody (1:2000, 4370, CST), anti-ERK antibody (1:1000, 4695, CST), anti-JUNB antibody (1:1000, C37F9, CST), anti-β-actin antibody (1:10000, 66009-1-Ig, Proteintech). Subsequently, membranes were detected by an enhanced chemiluminescence (Millipore) with a chemiluminescence imaging machine (Amersham Imager 600, GE) after incubated with HRP‐conjugated secondary antibodies (anti-mouse: 1:15000, ZB-2305, ZSGB-BIO; anti-rabbit: 1:8000, SA00001-2, Proteintech) at room temperature for another 2 h and quantiﬁed by Image J software.

**1.6 | Apoptosis analysis**

Primary chondrocytes were harvested after treated and stained by binding buffer with Annexin V – FITC and Propidium Iodide (PI) (88-8005, Invitrogen, USA) according to the product instructions. Flow cytometry (FACScalibur, Becton Dickinson (BD), USA) and Cell quest software (BD) were used to analyze chondrocytes that cells with Annexin V+/PI- were confirmed as early apoptosis, while Annexin V+/PI+ were late apoptosis.

**1.7 | Histology staining, immunofluorescence, and immunohistochemistry**

Patient cartilage and the whole left knee rat joints were fixed for 3 d in 4% paraformaldehyde at 26 °C and decalcified in 15% EDTA (pH 7.4) for 21 d at 37 °C. Tissues were then embedded in paraffin after dehydration with graded ethanol series and vitrification with xylene. Paraffinized tissues were sectioned (3 μm) continuously in sagittal plane. Sagittal sections were stained with Alcian Blue, Safranin O, or Toluidine Blue or IHC analysis for collagen II (1:100 for IHC; E-AB-70208; Elabscience) and FBXO21 (1:100 for IHC; Ab179818; Abcam, UK) according to the manufacturer's instruction was conducted after deparaffinization with xylene and hydration with graded ethanol series. The degree of cartilage degeneration was evaluated according to OARSI and Mankin scores (Gerwin, Bendele, Glasson, & Carlson, 2010; Pritzker et al., 2006; van der Sluijs et al., 1992). All images were scored by two researchers in a blinded manner.

Primary chondrocytes were seeded on cell climbing slides and washed three times with PBS after treatment with recombinant rat IL-1β for 12 h (0, 5, 10, or 20 ng/ml). Then, they were washed thrice with PBS after fixing in 4% paraformaldehyde for 30 min and 0.5% Triton-100 for 20 min. Next, they were blocked non-speciﬁc antigens by goat serum (ZSGB-BIO, China) and incubated with rabbit monoclonal anti-FBXO21 antibody (1:75 for immunofluorescence; Ab179818; Abcam) or rabbit polyclonal anti-collagen II (COL2A1) antibody (1:200 for IHC; E-AB-70208; Elabscience) overnight at 4°C.

For immunofluorescence, slides were incubated with fluorescent secondary antibodies (1:666; 4412; CST, USA) for 4 h and with DAPI (Solarbio) for 7 min in the dark at room temperature, followed by three PBS washes. They were imaged using a two-photon fluorescence microscope (Zeiss, Germany). For IHC, slides were sequentially treated with biotinylated secondary antibody, streptavidin/horseradish peroxidase, diaminobenzidine (DAB), and hematoxylin following the manufacturer's instruction (ZSGB-BIO). Confocal microscopy (Nikon, Japan) was performed, and images were quantiﬁed using Image J (National Institutes of Health, Bethesda, MD, USA).

**REFERENCES**

Gerwin, N., Bendele, A. M., Glasson, S., & Carlson, C. S. (2010). The OARSI histopathology initiative - recommendations for histological assessments of osteoarthritis in the rat. *Osteoarthritis Cartilage, 18 Suppl 3*, S24-34. doi:10.1016/j.joca.2010.05.030

Pritzker, K. P., Gay, S., Jimenez, S. A., Ostergaard, K., Pelletier, J. P., Revell, P. A., . . . van den Berg, W. B. (2006). Osteoarthritis cartilage histopathology: grading and staging. *Osteoarthritis Cartilage, 14*(1), 13-29. doi:10.1016/j.joca.2005.07.014

van der Sluijs, J. A., Geesink, R. G., van der Linden, A. J., Bulstra, S. K., Kuyer, R., & Drukker, J. (1992). The reliability of the Mankin score for osteoarthritis. *J Orthop Res, 10*(1), 58-61. doi:10.1002/jor.1100100107

**2 | TABLES**

**TABLE S1**: Spearman correlation analysis between FBXO21 expression and the baseline characteristics patients with osteoarthritis (*n* = 24)

| **Variables** | **FBXO21 expression** | |
| --- | --- | --- |
|  | **Spearman** | **p value** |
| Age | 0.24 | 0.258 |
| Gender | -0.097 | 0.652 |
| Weight | -0.147 | 0.493 |
| Height | 0.47 | 0.02 |
| Body Mass Index | 0.562 | 0.004 |
| Obesity gradation | 0.502 | 0.012 |
| Kellgren-Lawrence Gradation | 0.634 | 0.001 |

Note: *p* < 0.05 considered significant.

**TABLE S2**: Primers sequences of FBXO21, COL2A1, MMP13 and β-actin in SD rats

| **Name** | **Forward primer (5ʹ → 3ʹ)** | **Reverse primer (5ʹ → 3ʹ)** |
| --- | --- | --- |
| FBXO21 | TTTGGGAAGGGCAAGCAACT | CTGGTCAATGCCTTCCCTCT |
| COL2A1 | GCCAGGATGCCCGAAAATTAG | CCCTCTCTCCCTTGTCACCAC |
| MMP13 | TGAGTTTGCAGAGCACTACTTG | CAGGCACTCCACATCTTGGT |
| β-actin | TGTCACCAACTGGGACGATA | GGGGTGTTGAAGGTCTCAAA |

**TABLE S3**: Potential FBXO21-interacting proteins in chondrocytes by mass spectrometry

**Gene Name**

Kinesin-like protein

Proteasome 26S non-ATPase subunit 2 variant (Fragment)

Tyrosine-protein kinase receptor

Plastin-2

RNA-binding motif protein, X chromosome

T-complex protein 1 subunit theta

Caveolin

Tyrosine-protein kinase receptor

Cold shock domain containing E1, RNA-binding, isoform CRA_a

Very-long-chain (3R)-3-hydroxyacyl-CoA dehydratase

Cingulin-like protein 1

Nuclear pore complex protein Nup205

Fatty acid synthase

Nucleophosmin

Proliferating cell nuclear antigen

Elongation factor 1-gamma

Dystonin

L-lactate dehydrogenase B chain

Kelch-like protein 22

Heterochromatin protein 1-binding protein 3

Zinc finger protein 185

Isocitrate dehydrogenase [NADP]

Aspartyl/asparaginyl beta-hydroxylase

26S proteasome regulatory subunit 7

Sodium/potassium-transporting ATPase subunit alpha-1

Bleomycin hydrolase

Coatomer subunit alpha

Dolichyl-diphosphooligosaccharide--protein glycosyltransferase subunit 1

MICOS complex subunit MIC60

CD44 antigen

Collagen, type VI, alpha 3

Caldesmon

Transcription elongation factor SPT6

Twinfilin-1

TATA-binding protein-associated factor 2N

Proteasome 26S ATPase subunit 1 variant (Fragment)

Transketolase

NF110b

Serine/threonine-protein phosphatase PP1-alpha catalytic subunit

Serine/arginine-rich-splicing factor 1

Catenin beta-1

Nucleolar RNA helicase 2

Triosephosphate isomerase

ADP-ribosylation factor 4

PRKC apoptosis WT1 regulator protein

RAB1B protein

Serine/arginine-rich splicing factor 7

Kinectin 1 (Kinesin receptor), isoform CRA_a

Serine/threonine-protein phosphatase PGAM5, mitochondrial

Chaperonin containing TCP1, subunit 6A (Zeta 1), isoform CRA_a

Chromosome 7 open reading frame 24

Thioredoxin

Prolyl 4-hydroxylase subunit alpha-1

Protein ARPC4-TTLL3

Guanine nucleotide-binding protein G(s) subunit alpha isoforms Xlas

rRNA/tRNA 2'-O-methyltransferase fibrillarin-like protein 1

AP complex subunit beta

Truncated profilaggrin

ELAV-like protein 1

Chaperonin containing TCP1, subunit 2 (Beta), isoform CRA_b

Clathrin heavy chain

Extended synaptotagmin-2

Structural maintenance of chromosomes protein 4

Transcriptional activator protein Pur-beta

UHRF1-binding protein 1-like

GTP-binding protein Di-Ras2

Eukaryotic translation initiation factor 3 subunit L

Trifunctional enzyme subunit beta, mitochondrial

Talin-1

Aspartate--tRNA ligase, cytoplasmic

Prohibitin-2

DNA helicase

CDSN

Palladin

Lysosomal-associated membrane protein 1, isoform CRA_a

Proteasome subunit beta

Collagen alpha-1(I) chain

Very-long-chain enoyl-CoA reductase

Ubiquitin-like modifier-activating enzyme 1

T-complex protein 1 subunit gamma (Fragment)

Alpha-1,4 glucan phosphorylase (Fragment)

Kallikrein 7 (Chymotryptic, stratum corneum), isoform CRA_b

Serpin peptidase inhibitor, clade E (Nexin, plasminogen activator inhibitor type 1), member 1, isoform CRA_b

Tight junction protein ZO-2

Glucose-6-phosphate 1-dehydrogenase

Rab GDP dissociation inhibitor

Lysosome-associated membrane glycoprotein 2

Fragile X mental retardation autosomal homolog variant p2K

Reticulon-4

Isoleucyl-tRNA synthetase, cytoplasmic variant (Fragment)

Sphingosine-1-phosphate lyase 1

Voltage-dependent anion channel 2, isoform CRA_a

KN motif and ankyrin repeat domain-containing protein 2

Vesicle-trafficking protein SEC22b

Cytoplasmic FMR1-interacting protein 2

T-complex protein 1 subunit delta

DNA-dependent protein kinase catalytic subunit

ACLY variant protein (Fragment)

Eukaryotic translation initiation factor 2 subunit 1

Lamin-B1

Myoferlin

Calreticulin, isoform CRA_b

26S proteasome non-ATPase regulatory subunit 12

Histone-lysine N-methyltransferase EHMT2

Rho-associated protein kinase

Transcriptional adapter 2-beta

Delta (24)-sterol reductase

Mitogen-activated protein kinase

LUC7-like isoform b variant (Fragment)

EH-domain containing 2, isoform CRA_a

Note: IgG was negative control compared with FBXO21, FBXO21 minus IgG was the result.

**TABLE S4**: Predicted JUNB binding sites on FBXO21 promoter

| **Name** | **Forward primer (5ʹ → 3ʹ)** | **Reverse primer (5ʹ → 3ʹ)** |
| --- | --- | --- |
| Control PCR1 | AGCGTGGGCAACAGAGTG | CCTGGGTGACAGAACAAGA |
| FBXO21 PCR2 | AGCTGGTTAGACTATCGC | CCACTAAATCTGAAGGGA |
| FBXO21 PCR3 | GAGACTACCCTCCCAATC | AAGGCAAAGTTCTTCCAG |
| FBXO21 PCR4 | ACAAGCAAAAGTCAAGTGG | GTTGGTCAGGCTGGTCTC |

**3 | FIGURE LEGENDS**

**FIGURE S1** Expression of FBXO21 is upregulated in the damaged area of articular cartilage in patients with knee osteoarthritis (OA) who underwent total knee replacement. **(a)** Relationship between age, gender and FBXO21 expression in the damaged area represented by a Sankey diagram. **(b)** Relationship between obesity gradation, Kellgren-Lawrence gradation and FBXO21 expression in the damaged area represented by a Sankey diagram. Underweight: BMI < 18.5, Normal weight: 18.5 ≤ BMI < 24, Overweight: 24 ≤ BMI < 28, Overweight: BMI ≥ 28.

**FIGURE S2** FBXO21 accumulated in rat articular cartilage and chondrocytes with OA. **(a)** Immunofluorescence analysis of FBXO21 (Green) in chondrocytes stimulated with interleukin (IL)-1ꞵ. **(b)** Immunoblotting (upper) results of FBXO21, COL2A1, and MMP13 and the quantification (lower) of FBXO21 with ꞵ-actin as the endogenous control in SW1353 cells stimulated with IL-1ꞵ, tumor necrosis factor (TNF)-α, and lipopolysaccharide (LPS). Data are presented as the mean ± SD; ns: not significant, **p* < 0.05, ***p* < 0.01, ****p* < 0.001, *****p* < 0.0001.

**FIGURE S3** FBXO21 knockdown suppresses OA-related degeneration in MIA-treated rats and IL-1ꞵ-treated rat chondrocytes. **(a)** Macroscopic score of gross imaging for MIA-2W OA model of SD rat after transfected with FBXO21 knockdown adenovirus divided into KD-Ad-shRNA-NC (KD-NC), KD-Ad-shRNA-FBXO21-01 (KD-01) and KD-Ad-shRNA-FBXO21-02 (KD-02). NC: negative control. **(b)** Makin score and Osteoarthritis Research Society International (OARSI) score after knockdown of FBXO21 based on Toluidine Blue and Safranin O staining. Ctrl: Saline Controls, 2W: MIA-2W. Quantitative real time (qRT)-PCR analysis of FBXO21, COL2A1, and MMP13, with ꞵ-actin as the endogenous control after knockdown of FBXO21 in **(c) rat** knee cartilage of and **(e)** rat chondrocytes. 20: 20ng/ml of interleukin (IL)-1ꞵ. Quantification for relative protein level of FBXO21, COL2A1, Aggrecan, MMP3, MMP13, LC3 II/I, Beclin1, Bcl2/Bax with ꞵ-actin as the endogenous control after knockdown of FBXO21 in **(d) rat** knee cartilage and **(f)** rat chondrocytes. **(g)** The Number of GFP and mRFP dots per cell in chondrocytes after knockdown of FBXO21. **(h)** The Number of autophagosomes in chondrocytes after knockdown of FBXO21. **(i)** Flow cytometry analysis and **(j)** quantification in chondrocytes after knockdown of FBXO21. Apoptosis rate was equated to percent of right lower quadrant (early apoptosis) and right upper quadrant (late apoptosis). Data are presented as the mean ± SD; ns: not significant, **p* < 0.05, ***p* < 0.01, ****p* < 0.001, *****p* < 0.0001.

**FIGURE S4** FBXO21 overexpression promotes OA-related degeneration in MIA-treated rats and IL-1ꞵ-treated rat chondrocytes. **(a)** Safranin O staining of SD rat knee joints after knockdown and overexpression of FBXO21. Boxed regions were presented at higher magnification. Ctrl: Saline Controls, 2W: MIA-2W. **(b)** Makin score and Osteoarthritis Research Society International (OARSI) score for MIA-2W OA model of SD rat after transfected with FBXO21 overexpression (OE) adenovirus (Ad) divided into OE-Ad-NC (OE-NC) and OE-Ad-FBXO21-3xflag (OE-FBXO21) based on Toluidine Blue and Safranin O staining. NC: negative control. Quantification for relative protein level of FBXO21, COL2A1, Aggrecan, MMP3, MMP13, LC3 II/I, Beclin1, Bcl2/Bax with ꞵ-actin as the endogenous control after overexpression of FBXO21 in **(c) rat** knee cartilage and **(d)** rat chondrocytes. 2W: MIA-2W, 20: 20ng/ml of interleukin (IL)-1ꞵ. **(e)** The Number of autophagosomes in chondrocytes after overexpression of FBXO21. **(f)** Apoptosis rate was equated to percent of right lower quadrant (early apoptosis) and right upper quadrant (late apoptosis). Data are presented as the mean ± SD; ns: not significant, **p* < 0.05, ***p* < 0.01, ****p* < 0.001, *****p* < 0.0001.

**FIGURE S5** JUNB accelerates OA-related degeneration by promoting FBXO21 expression. **(a)** Predictive transcription factors (left) of FBXO21 (Homo sapiens) by JASPAR database that track score ≥ 550. de novo motifs of JUNB (right). **(b)** Quantification for relative protein level of JUNB in undamaged and damaged areas (*n* = 24). **(c)** Relationship between age, gender and JUNB expression in the damaged area represented a by Sankey diagram. **(d)** Linear regression analysis of the expression of JUNB and FBXO21 in the undamaged area in patients with knee OA (*n* = 24). **(e)** Quantification for relative protein level of JUNB, FBXO21, COL2A1, Aggrecan, MMP3, MMP13, LC3 II/I, Beclin1 and Bcl2/Bax in SW1353 cells transfected with FBXO21-pEX3 overexpression plasmid (OE-FBXO21) and JUNB knockdown plasmid divided into KD-shRNA-NC (KD-NC) and KD-shRNA-JUNB (KD-JUNB) with ꞵ-actin as the endogenous control. **(f)** The Number of GFP and mRFP dots per cell in SW1353 cells. **(g)** Flow cytometry analysis and **(h)** quantification in SW1353 cells after knockdown of JUNB and overexpression of FBXO21. Data are presented as the mean ± SD; ns: not significant, **p* < 0.05, ***p* < 0.01, ****p* < 0.001, *****p* < 0.0001.

**FIGURE S6** JUNB accelerated OA-related degeneration by promoting FBXO21 expression. **(a)** Immunoblotting and **(b)** quantification for relative protein level of JUNB, FBXO21, COL2A1, Aggrecan, MMP3, MMP13, LC3 II/I, Beclin1 and Bcl2/Bax in rat chondrocytes transfected with FBXO21 overexpression adenovirus (OE-FBXO21) and JUNB knockdown adenovirus divided into KD-Ad-shRNA-NC (KD-NC) and KD-Ad-shRNA-JUNB (KD-JUNB) with ꞵ-actin as the endogenous control. Data are presented as the mean ± SD; ns: not significant, **p* < 0.05, ***p* < 0.01, ****p* < 0.001, *****p* < 0.0001.
